# The Effect of Music Tempo on Fatigue Perception at Different Exercise Intensities

**DOI:** 10.3390/ijerph19073869

**Published:** 2022-03-24

**Authors:** Jianfeng Wu, Lingyan Zhang, Hongchun Yang, Chunfu Lu, Lu Jiang, Yuyun Chen

**Affiliations:** 1 Industrial Design and Research Institute, Zhejiang University of Technology, Hangzhou 310023, China; jianfw@126.com (J.W.); yhc2016@zjut.edu.cn (H.Y.); 2 School of Design and Architecture, Zhejiang University of Technology, Hangzhou 310023, China; lingyanzh@outlook.com (L.Z.); jl01103149@alibaba-inc.com (L.J.); 2111915006@zjut.edu.cn (Y.C.)

**Keywords:** running, music tempo, exercise intensity, fatigue perception, heart rate, surface electromyographic signals, median frequency

## Abstract

Background: This study aimed to clarify the effect of music tempo on runners’ perception of fatigue at different exercise intensities and while listening to music of different tempos through running experiments. Methods: This study used a within-subject two-factor experimental design with music tempo (fast music, slow music, no music) and exercise intensity (high intensity, low intensity) as independent variables and the time to fatigue perception (TFP), the difference in heart rate (HR) and the difference in the median frequency (MF) of surface electromyography (sEMG) signals as observation indexes. Eighteen participants completed a total of 108 sets of running experiments. Results: (1) The main effect of music tempo on the TFP was significant (*p* < 0.001). (2) The main effect of exercise intensity on the TFP was significant (*p* < 0.001), and the main effect on the difference in HR was significant (*p* < 0.001). (3) The interaction effect of music tempo and exercise intensity on the TFP was significant (*p* < 0.05). Conclusions: Exercisers’ subjective perception of fatigue was affected by music tempo and the interaction between music tempo and exercise intensity, and exercisers’ objective fatigue perception was influenced mostly by exercise intensity. The findings of this study provide guidance for runners’ choice of music at different intensities of exercise. Whether it is low-intensity exercise or high-intensity exercise, listening to fast music while exercising can help runners perform better mentally and physically during their runs.

## 1. Introduction

Runners’ fatigue perception varies while running. Fatigue perception refers to the subjective intensity of perception of tension, discomfort and fatigue during physical exercise [1]. Exercise fatigue usually manifests as soreness in muscles, an increased heart rate and decreased cognitive performance [2,3]. Although the continuous accumulation of fatigue will affect people’s exercise intentions and performance [4] and excessive fatigue will lead to physical injury [5,6], appropriate exercise fatigue can improve physical performance [7]. Therefore, to achieve a better fitness effect, runners usually adjust their exercise intensity continuously according to their subjectively perceived state after perceiving exercise fatigue during exercise [8]. Listening to music to reduce the sense of boredom and fatigue during running and to enhance motivation has become a common behavior during running [9].

In terms of whether music interferes with the perception of fatigue in exercisers, there is a consensus among academics that the effects of music can be explained from three perspectives: emotional regulation, attention diversion and fatigue recovery. Emotion plays an important role in regulating motivation in sports. Music can cause exercisers to experience positive emotions during exercise [10], alleviate their perception of fatigue during running and enhance the pleasure and sense of participation in running [11]. Using parallel information processing theory, Rejeski points out that the bandwidth of human attention processing narrows during exercise. In the absence of the external stimuli of music, runners may pay more attention to their running behaviors and feelings, thus increasing their perception of fatigue, while in conditions with music, runners shift their attention from unpleasant physical feelings to music, reducing their perception of fatigue and other negative feelings [12]. It has also been pointed out that the tempo of music can influence the internal movement tempo of the body [13], and music with a good tempo can induce specific movement patterns [14] and enhance the arousal level of the exerciser, raising the threshold of fatigue perception and speeding up recovery from exercise fatigue [15,16].

Further studies have shown that exercise fatigue perception is influenced by both music tempo and exercise intensity. Different tempos stimulate different emotional states in listeners, resulting in different effects on runners’ fatigue perception [17]. Exercise intensity is another frequently discussed influencing factor. It has been argued that music can play a role in reducing the perception of fatigue at different exercise intensities [18]. However, some scholars believe that music only plays a regulatory role at specific intensities [19]. Subsequently, many scholars have conducted studies on music tempo and exercise intensity, trying to find the optimal combination of exercise intensity and music tempo for modulating fatigue perception. For example, Maddigan et al. concluded that fast music could improve the performance of exercisers during high-intensity exercise and reduce fatigue perception [20], while Yamamoto et al. found that neither fast music nor slow music changed the average power output of exercisers during high-intensity exercise [11], i.e., different music tempos did not disperse individuals’ perception of fatigue. Some scholars have also discussed how movement perception is influenced by the interaction of music tempo and movement-related fatigue, but the key lies in the music tempo, the consistency of exercisers’ preference and in the music and movement tempos being synchronous or asynchronous. No study compared music tempo to runner fatigue perception at different intensities of exercise. In addition, some scholars have discussed whether exercisers’ fatigue perception is affected by the interaction between music tempo and exercise intensity, but the studies focused on the consistency of music tempo with exercisers’ preference [21] or the influence of music tempo being synchronous or asynchronous with exercise tempo [22], and there is no study comparing the effect of music tempo on runners’ fatigue perception at different exercise intensities for the time being.

The aforementioned studies provide references for the positive effects of music on exercise fatigue perception, but there is still some space for exploration on how to choose the appropriate tempo of music for different intensities of exercise. For example, how does musical tempo affect fatigue perception of exercisers during different intensities of exercise? Is there an interaction effect between music tempo and exercise intensity on runners’ fatigue perception? All these problems need our attention, and the effects of music tempo on fatigue perception at different exercise intensities still need to be further explored.

To clarify the effect of music tempo on runners’ fatigue perception at different exercise intensities, this study conducted experiments on running and analyzed and discussed the changes in runners’ fatigue perception at different exercise intensities and with different music tempo conditions by evaluating runners’ fatigue perception. The results provide guidance for individual fitness practitioners choosing music to listen to during exercise at different intensities.

## 2. Methods

### 2.1. Subjects

We conducted an a priori analysis of the required sample size in the study using G*power, with the presumption of the presence of a medium effect size of *f* = 0.25 [23], a statistical test power = 0.8 and a significance level α = 0.05. The results of the analysis indicated that a sample size of 18 would be sufficient to achieve a medium effect size interaction effect. To obtain more generalizable research conclusions, the experimental subjects were ordinary college students who did not have regular fitness habits and were not guided by scientific theories or methodological knowledge of running fitness. They had no diseases of the muscular, skeletal, respiratory or cardiovascular systems. They were not allergic to alcohol and had no cuts or scratches on the thigh muscles. All subjects had no significant differences in physical indicators, were between 20 and 30 years old, 170 and 180 cm in height and 55 and 75 kg.

A total of 18 healthy males were recruited as experimental subjects after the above rigorous screening. The basic information of the experimental subjects is shown in Table 1. Participants were required to confirm compliance with the following requirements prior to each experiment: (1) Rest at least 8 h the night before each experiment and refrain from participating in any other physical activity other than the exercise tasks of this experiment to prevent cardiopulmonary or muscle function injury or abnormalities. (2) Maintain a previously habitual daily diet and ensure that the experiment is performed at least one hour after the meal, and avoid eating, drinking alcohol or consuming excessive water within one hour prior to the experiment.

### 2.2. Experimental Design

The experiment was a two-factor within-subject experimental design with the independent variables being music tempo (fast tempo, slow tempo, no music) and exercise intensity (high intensity, low intensity). The two independent variables led to a total of six experimental protocols. The dependent variables were the time to fatigue perception (TFP), heart rate (HR) and the median frequency (MF) of surface EMG signals. For each of the three music conditions, the experiment was completed for high- and low-intensity running. To prevent interference between different groups of experiments, the interval between each running experiment was 48 h for each subject, and the running experiments for each subject were scheduled at the same time of day (from 9:00 a.m. to 11:00 a.m.) to control for possible effects of diurnal patterns [24]. Fresh air circulation in the room was maintained during the experiment, and the ambient temperature was kept at 23 ± 2 °C to reduce the environmental load, promote normal body heat dissipation in the subjects during the experiment and to avoid electrode shedding or short circuits caused by excessive sweating. Each participant signed an informed consent form for the experiment before participating. The study was conducted in accordance with the Declaration of Helsinki and approved by the Ethics Committee of the Industrial Design Institute of Zhejiang University of Technology (protocol code 0903/2021, date of approval 15 September 2021) (Supplementary Materials Files S1 and S2).

### 2.3. Independent Variable

#### 2.3.1. Music Tempo

Music can be divided into fast tempo music and slow tempo music according to the speed of the tempo. The beat of fast tempo music is 150–160 bpm, and the beat is strong. The beat of slow tempo music is 90–100 bpm, and such music is characterized by a narrow range, soft sound and soothing changes [25]. In addition to the requirements of music tempo, this study also followed the following principles of song selection: (1) The songs chosen had a cheerful music style and obvious tempo and were able to induce positive emotions in the subjects. (2) Music without any lyrics was selected to avoid the interference of lyric content. (3) Songs with complex tunes were excluded. According to the principle of music selection, 15 pieces of fast tempo music and 15 pieces of slow tempo music were selected.

All subjects who participated in the experiment were invited to score the valence and arousal effect of each piece of music, and the music with higher validity and arousal effect among the fast music and slow music was selected for inclusion in the final song list. Finally, 15 pieces of music were obtained for the running experiment. Among them, 9 were slow tempo pieces and 6 were fast tempo music pieces. Details of the selected music are shown in Table 2.

#### 2.3.2. Exercise Intensity

Exercise intensity refers to the amount of exercise an individual can perform per unit of time and is a very important indicator in physical exercise and training. The American College of Sports Medicine suggests that adults should exercise at an exercise intensity of 50–85% of heart rate reserve (%HRR) to improve their cardiopulmonary function [26]. In view of the fact that this study included people who usually exercise less, the exercise intensity during the experiment was within the recommended range of %HRR, with 50–60% HRR for low exercise intensity and 70–80% HRR for high exercise intensity.

To facilitate the experiment, the mean treadmill speed was adjusted according to the %HRR interval to explore the relationship between %HRR and exercise intensity and determine the treadmill speed to use for the running experiment [27]. Half of the subjects (7 in total) were randomly selected to participate in the treadmill speed determination experiment. Each subject was required to perform two separate running experiments at 50–60% HRR and 70–80% HRR, respectively. To avoid the effect of fatigue, the two running experiments were separated by more than 24 h. The experimental steps were as follows: (1) the resting HR of the subjects was measured to determine the target HR range; (2) the subjects wore an HR belt and then performed a 3 min warm-up exercise on the treadmill; (3) an incremental running experiment was conducted, in which the subjects started running at a speed of 5.5 km/h and the speed was increased by 0.5 km/h every 2 min; (4) after each subject reached the upper limit of the target HR, the subject ran continuously at that speed and stopped after 5 min. During the 5 min, the change in target HR of the subjects was observed, and the HR of the subjects was kept at the upper limit of the target HR range by increasing or decreasing the speed of the treadmill as appropriate. The target HR was calculated by Equation (1):

Target HR = target intensity %HRR × (HRmax − resting HR) + resting HR(1)

Based on the average speed of the treadmill while the subjects were in the target HR range, the treadmill speed corresponding to low-intensity exercise (50–60% HRR) for this experiment was 7 km/h, and the treadmill speed corresponding to high-intensity exercise (70–80% HRR) was 9 km/h.

#### 2.3.3. Music Tempo with Exercise Intensity

The subjects performed constant-load running exercises on a home motorized treadmill in the general fitness mode at different intensities under the conditions of music or no music. A total of 6 exercise regimens were used for subjects within the 3 × 2 group: no music × low intensity, no music × high intensity, slow tempo × low intensity, slow tempo × high intensity, fast tempo × low intensity, fast tempo × high intensity. To prevent the interference of order effects, the Latin square design method was used to arrange the experimental order in this experiment.

### 2.4. Dependent Variable

#### 2.4.1. Time to Fatigue Perception

In this study, Borg’s scale for rating of perceived exertion (RPE) was used to measure the subjective exertion of exercisers during running. Scores on the RPE scale ranged from 6 (no exertion at all) to 20 (exercise limit), as shown in Table 3. The RPE scale is a subjective assessment of an individual’s perception of muscular exertion, physical tension, discomfort or fatigue during exercise and reflects the individual’s perception of his or her fatigue state [28]. When the RPE reaches 15, the exerciser shows shortness of breath and significant muscle fatigue. Therefore, to ensure the safety of the experiment, RPE = 15 was chosen as the index for the experiment. Running was stopped when the RPE value reported by the subject reached 15, and the time from the start of running to the time when the RPE reached 15, i.e., the time to fatigue perception, was recorded.

#### 2.4.2. Instantaneous HR

HR can be used to objectively evaluate exercise fatigue and is the easiest indicator to use to assess the intensity of current exercise and exercise fatigue [29]. Exercise fatigue leads to a decrease in the HR regulation function, HR increases with fatigue and HR is dynamic during exercise. In this study, the difference in HR before and after running was used to characterize the degree of fatigue of exercisers. Two heart rate measurements were taken in each set of experiments; the first time the subject’s resting HR was measured before the run. The second time was measured during the running process, where the subject was wearing a heart rate belt, and we collected the HR signal of the subject during the whole running process. However, in the data processing, only the heart rate signal of the subject 5 s before the end of the run was selected and averaged as an indicator of the immediate post-run HR.

#### 2.4.3. Surface Electromyography Signal

Electromyography (EMG) signals are indicative of biological electrical signals generated by the contraction of human muscles. Surface EMG (sEMG) measures the comprehensive electrical effect of the conduction of human muscle electrical signals that can be sensed on the skin surface [30]. The sEMG signal is commonly used for the evaluation of neuromuscular function because of its real-time, sensitive and flexible characteristics. It has important practical and research value in the fields of sports science, clinical medicine, ergonomics, etc. There have been many studies linking sEMG signals to fatigue [31]. The sEMG uses the surface electrode bipolar conduction method, and the derived EMG signal is increased by a signal amplifier and then enters a converter for signal conversion and storage in a computer. It has been shown that the frequency domain index of the sEMG signal is more sensitive to the muscle fatigue state of runners [32]. Frequency domain analysis mainly relies on fast Fourier transformation (FFT) to obtain the frequency spectrum or power spectrum of the EMG signal to reflect the variation of the EMG signal in the frequency dimension [33]. The median frequency (MF) is one of the most commonly used indicators in the frequency domain analysis of surface EMG signals, and the MF value is more stable for assessing the muscle fatigue state [34,35]. MF refers to the middle value of muscle fiber discharge frequency during skeletal muscle contraction and high frequency discharge is the main expression of excitation of fast muscle fibers, while slow muscle fibers are dominated by low-frequency potential activity [36]. Therefore, the difference in static MF before and after running was used as the index of local muscle fatigue in this study. The formula for MF is shown in Equation (2):

(2) $$\mathrm{MF}=\frac{1}{2}\int_{0}^{\infty }PSD(f)df$$

where *PSD(f)* is the myoelectric power spectral density.

**Figure 1:**
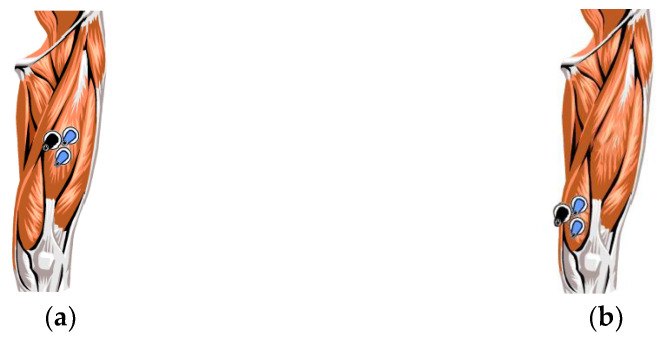
Muscle and electrode location. (**a**) Shows the electrode positions for the RF, (**b**) shows the electrode positions for the VM.

Running mainly mobilizes the leg muscle groups; therefore, in this experiment, the sEMG signals of the local muscles in the runners’ legs were collected, and the rectus femoris (RF) and vastus medialis (VM), which display obvious changes in sEMG signals and are very stable during movement changes, were examined [37]. The electrode positions for the RF and VM are shown in Figure 1. Figure 1a shows the electrode positions for the RF, Figure 1b shows the electrode positions for the VM. The black electrode is the reference electrode.

The RF and VM sEMG signals of the subject’s dominant leg were collected. The subject was first asked to perform thigh flexion and extension movements to find the target muscle location. Once the target muscle was found, the location of the target muscle was marked [38]. Subsequently, cotton dipped in alcohol was used to clean the skin of surface dirt and remove sweat, sebum and other impurities on the skin surface. The electrodes were pasted after the skin was dry, and surface hairs were removed if necessary [39]. The purpose of taking the above measures was to reduce the impedance effect of the skin, enhance the adhesion of the electrode patch to the skin and obtain the best recording effect. Following the surface EMG for non-invasive assessment of muscles (SENIAM) guidelines, bipolar sEMG electrodes were placed along the longitudinal midline of the muscle (in the direction of the muscle fibers) on the muscle abdomen at the muscle–tendon junction, and the point spacing of the electrode patch was 2–3 cm [40,41]. After placement of the sensor and the reference electrode, a test was performed to determine whether the electrodes were placed properly on the muscle and connected to the equipment so that a reliable sEMG signal could be recorded [42]. EMG signal acquisition software (Acknowledge4.2, Biopac) was connected to the sensor, and then the subject was asked to perform flexion and extension movements. It was then observed whether the corresponding EMG signal in the interface of the acquisition software produced obvious changes. If the signal did not show obvious changes, or the signal showed abnormal values, the muscle selection position was further calibrated, and the electrode patch was checked to determine whether the connection with skin was firm [38]. Testing continued until the signal was stable. The above steps were followed before each acquisition of sEMG signals. The electrodes used for testing were Ag/AgCl electrocardiographic electrodes. The EMG100c signal amplifier was then secured to the subject’s lower leg with a strap.

### 2.5. Experimental Apparatus

The experimental apparatus included a treadmill, a pair of Bluetooth headphones, a set of barbell pieces with different weights, an HR belt, an RPE scale and an MP150 telemetry physical recorder and its accessories. The treadmill had a rated power of 1100 W and a belt area of 1350 × 480 mm, which was used to provide running conditions with a constant load. Airpod Bluetooth headphones were used to play music at a constant volume of 50% of the maximum volume, or approximately 65 dB. Barbells were hung on the legs before and after running to collect sEMG signals during static muscle exertion. A Polar HR monitor (Polar Electro Oy, Kempele, Finland) with a sampling rate of 125 Hz was used to acquire the real-time HR of the subjects during running. Participants wore the Polar heart rate band on their chest, which synced with their iPhone via Bluetooth. The MP150 telemetry physiological recorder (Biopac Inc., Goleta, CA, USA) was used to acquire sEMG signals at a sampling rate of 2048 Hz, equipped with two EMG100c signal amplifiers and several disposable ECG electrode patches (Shanghai Huyou Medical Electrode Co., Ltd., Shangai, China). The electrodes were used to connect the target muscle to the EMG signal amplifier. The bipolar electrode (Ag/AgCl) was attached with a 10 mm diameter gel medium for reducing the impedance between the electrode and the skin. The size of the electrode patches was trimmed to 3 cm before use so that they could meet the electrode patch 2–3 cm spacing distance.

### 2.6. Experimental Procedure

**Figure 2:**
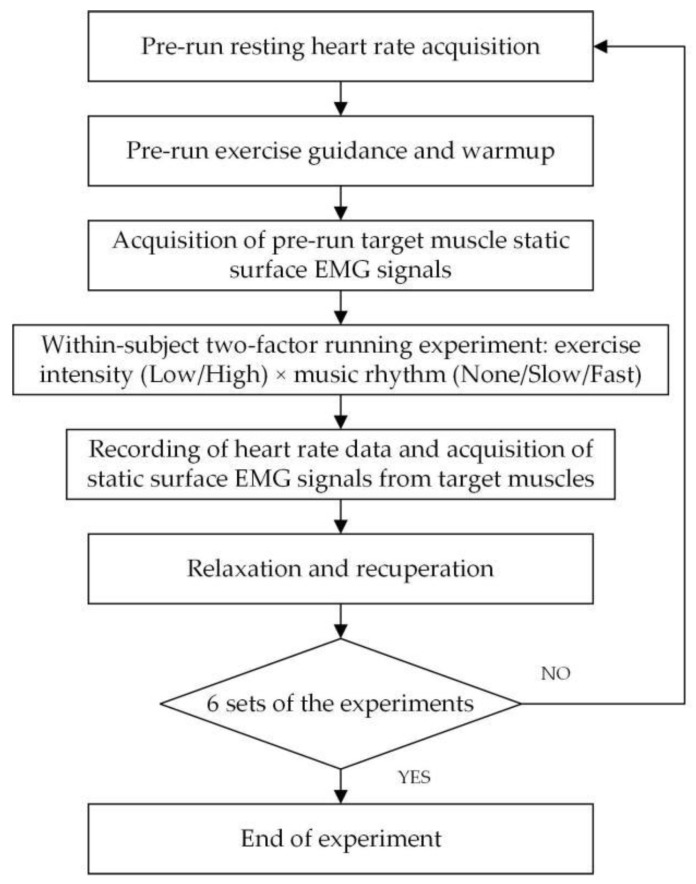
Experiment flow chart.

The experimental procedure for this study is shown in Figure 2. Each subject was required to follow the experimental procedure and complete a total of six sets of the running experiment.

#### 2.6.1. Acquisition of Resting HR

The HR of the subject was captured and recorded in real-time with an HR band. To exclude the influence of the initial state among different subjects, the resting HR of the subjects was collected before the experiment began. After the subjects sat in a comfortable position for 5 min, their HR was collected for 1 min, and the average value was calculated as the resting HR of the subjects [28].

#### 2.6.2. Pre-Run Safety Instructions and Warmup

Before the formal experiment started, the subjects were introduced to the experiment contents and procedures in detail and received instructions on the use of the treadmill, correct running posture, a breathing adjustment method that could be used during running and the use of the RPE scale to ensure the safety of the subjects and the smooth operation of the experiment. All subjects underwent a 5-min warmup exercise before the experiment to avoid the occurrence of muscle damage during the experiment.

#### 2.6.3. Acquisition of sEMG before Running

Static sEMG signals of the abovementioned muscles were collected before and after running to complement the instability of the sEMG signals during running [28]. A set of barbell pieces of different weights was prepared for hanging on the dominant leg before and after running to collect sEMG signals during static muscle exertion. Static sEMG signals were acquired as follows: the subject’s leg was subjected to a certain percentage of weight-bearing and knee extension so that the leg muscles were in a state of exertion, and then data were recorded for the RF and VM with an MP150 telemetry physical recorder for 30 s [43]. In this case, the weight-bearing level was determined based on the body weight of each subject: weight-bearing level = body weight (kg) × 10% [44]. The EMG amplitudes of the RF and VM on the dominant side of the leg were recorded. To ensure signal stability and data reliability, the middle 20 s of the 30-s static EMG signal were extracted, and the 20-s MF values were calculated with a 2048-point window.

**Figure 3:**
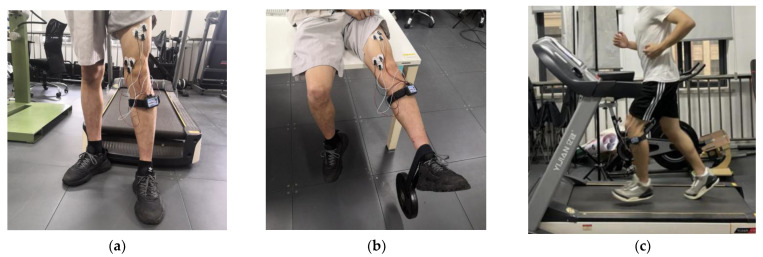
(**a**) The electrode position for the target muscles and the actual situation of wearing and using the instrument; (**b**) the setup for measuring sEMG signals when loading and stretching the knee; and (**c**) the running experimental procedure.

The experimental procedure is shown in Figure 3. Figure 3a shows the electrode positioning for the target muscle and the actual situation of wearing and using the instrument; Figure 3b shows the setup for measuring sEMG signals when loading and stretching the knee; and Figure 3c shows the running experimental procedure.

#### 2.6.4. Running Experiment and Data Collection

Each runner ran under low-intensity and high-intensity conditions, with no music, fast music and slow music. The subjects started exercising at 7 km/h for the low-intensity exercise condition and at 9 km/h for the high-intensity exercise condition. The subjects were observed and asked about their current fatigue at 1-min intervals, and the RPE values were recorded. The subjects stopped running when their subjective RPE reached 15. The time taken for the RPE value to reach 15 was recorded, and the HR signal was collected for 5 s before the end of the run. Immediately after the subjects stopped running, the weighted knee extension experiment was performed, and the sEMG signals of the RF and VM were collected from the subjects. Throughout the experiment, the experimenter observed the subjects’ movement status and ensured their safety.

After the experiment, the HR band, electrode pads and other experimental equipment were removed, and the subjects were asked if there was any discomfort and were instructed to perform appropriate stretching and recovery exercises to regulate HR recovery. Then, the next experiment was scheduled.

### 2.7. Data Processing and Analysis

All results are presented as group means and standard deviations. The normality of the data distribution was confirmed using the Shapiro–Wilk test. To determine the effect of the intervention on the dependent variable, a two-way analysis of variance (ANOVA) for repeated measures was used to calculate between-group differences. If the interaction between music tempo and exercise intensity was significant, Bonferroni post hoc tests were calculated. The effect size was calculated as partial eta squared (η^2^). The criteria for classifying Cohen d were as follows: small (0 < d < 0.5), medium (0.5 ≤ d < 0.8) and large (d ≥ 0.8) [23]. The significance level was 0.05. All statistical analyses were performed using SPSS 26.0 (SPSS Inc., Chicago, IL, USA).

## 3. Results

### 3.1. Examination of the Effects of Music Tempo and Exercise Intensity

A two-way repeated-measures ANOVA was conducted for TFP and the differences in HR and MF to determine whether there were main and interaction effects for music tempo and exercise intensity, and the results are shown in Table 4. The main effect of music tempo on TFP was significant (*p* < 0.001), but the main effects of the differences in HR and MF were not significant (*p* > 0.05). The main effect of exercise intensity on TFP and the difference in HR was significant (*p* < 0.001), but the main effect for the difference in MF was not significant (*p* > 0.05). The interaction effect of music tempo and exercise intensity was significant for TFP (*p* < 0.05) but not for the difference in HR or MF (*p* > 0.05).

### 3.2. Time to Fatigue Perception

**Figure 4:**
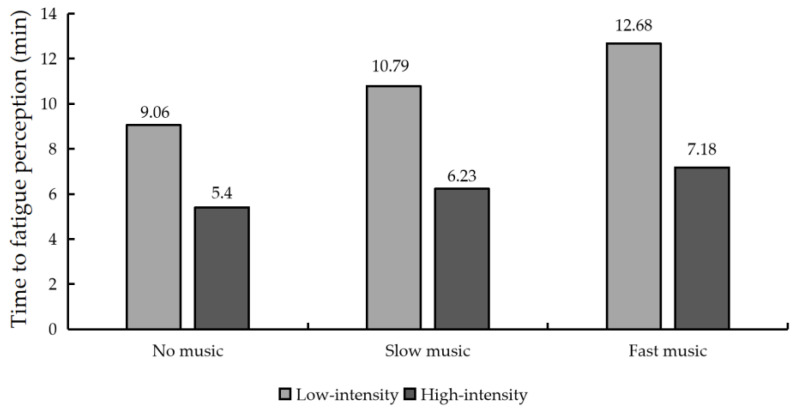
Time to fatigue perception for different experimental conditions.

The effects of music tempo on TFP at different exercise intensities are shown in Figure 4. The combined results show that the TFP of the group with music was significantly greater than that of the group without music. Specifically, in the low-intensity exercise experiment, the TFP in the no music condition (9.06 min) was shorter than the TFP in the slow music condition (10.79 min) and the fast music condition (12.68 min). During high-intensity exercise, the TFP in the no music condition (5.40 min) was shorter than the TFP in the slow tempo music condition (6.23 min) and the TFP in the fast tempo music condition (7.18 min).

Since the interaction effect of music tempo and exercise intensity on the TFP was significant, a further Bonferroni post hoc test was performed to show the results (Table 5). The results of the Bonferroni post hoc test showed that the difference in the effect on TFP between no music and slow music at different exercise intensities was statistically significant (*p* < 0.01), the difference in the effect on TFP between no music and fast music was statistically significant (*p* < 0.001) and the difference in the effect on TFP between slow music and fast music was statistically significant (*p* < 0.001).

### 3.3. HR Changes

**Figure 5:**
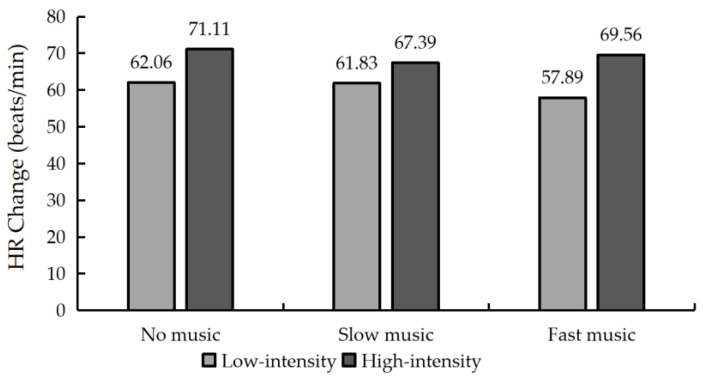
Differences in HR before and after running under different experimental conditions.

The effect of music tempo on the differences in HR at different exercise intensities is shown in Figure 5. As seen from the difference in HR between the before and after measurements, the difference in HR in the high-intensity exercise group was higher than that in the low-intensity group overall. This is evident in the following: during low-intensity exercise, the no music group had the highest HR variation (62.06 beats/min), followed by the slow music group (61.83 beats/min) and finally the fast music group (57.89 beats/min); during high-intensity exercise, the no music group had the highest HR variation (71.11 beats/min), followed by the fast music group (69.56 beats/min) and finally the slow music group (67.39 beats/min).

### 3.4. sEMG Changes

**Figure 6:**
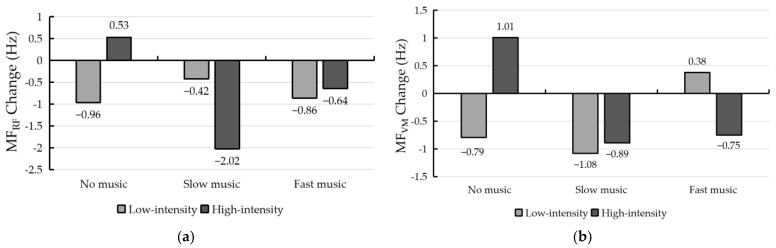
(**a**) MF differences of rectus femoris before and after running under different experimental conditions; (**b**) MF differences of vastus medialis before and after running under different experimental conditions.

The results of the MF difference of EMG signals of the RF and VM of the subjects under the six exercise protocols are shown in Figure 6a,b. From the figure, it can be seen that the MF difference values of both the RF and VM have two different directions of positive and negative results, failing to show a regular variation in surface EMG signal changes of the RF and VM.

## 4. Discussion

### 4.1. The Effect of Music Tempo on Runners’ Subjective Perception of Fatigue at Different Exercise Intensities

The TFP results indicate that music can effectively influence runners’ perception of fatigue during running, a finding that is consistent with previous studies [45,46]. The significant main effect of music tempo (*p* < 0.001) also confirms the positive effect of music on fatigue perception (0.50 ≤ effect size d < 0.8). The results show that music tempo had a moderate effect on the time of fatigue perception. This result is consistent with Nethery’s study, where listening to music reduced subjective fatigue induced by exercise at different exercise intensities [18]. The mechanism of the effect can be explained by attentional limitation theory and selective sensory filtering theory [47,48]. That is, listening to music during exercise can reduce the excitability of the sympathetic nervous system, thereby reducing subjective fatigue perception and enhancing exercise tolerance. This study further explored the influence of music tempo on the TFP at different exercise intensities. This also suggests that in the absence of musical stimuli, participants may be more focused on their efforts and feel fatigued more quickly.

The significant main effect of exercise intensity (*p* < 0.001) indicates that there was a significant difference between the effects of high and low exercise intensity on runners’ subjective perception of fatigue, and there was a moderate effect of exercise intensity on subjective fatigue perception (0.50 ≤ effect size d < 0.8), which is consistent with common knowledge about the positive correlation between exercise intensity and fatigue perception. Meanwhile, the significant interaction effect of music tempo and exercise intensity indicated that the effects of music tempo and exercise intensity (*p* < 0.05) on runners’ subjective perception of fatigue were influenced by each other, but the effect is small. However, the effect size result (0 < effect size d < 0.5) shows that music tempo and exercise intensity interacted to a lesser extent. The results of further post hoc analyses indicate that whether in high intensity or low intensity exercise, listening to fast music can prolong the running time and reduce the subjective fatigue of runners compared to listening to slow music. During high-intensity exercise, the effect of fast music and slow music on the TFP is significant (*p* < 0.001), indicating that fast music was to some extent more effective than slow music in reducing fatigue perception. This conclusion is also supported by previous studies, such as Cental’s study, which mentioned that listening to fast music increased overall exercise tolerance and the neuromuscular fatigue threshold [18]. Furthermore, post-experiment interviews with all subjects revealed that listening to fast music resulted in a more uplifted state of mind and a more positive mood, which effectively distracted them and reduced the perception of fatigue. Some subjects also believed that fast music made them unconsciously adjust their running pace, which helped to counteract the perception of fatigue. The combined results of the experiments show that fast music effectively reduced the subjective perception of fatigue at different exercise intensities in runners.

### 4.2. The Effect of Music Tempo on HR Differential at Different Exercise Intensities

The ANOVA results show that the difference in HR before and after running was only affected by the main effect of exercise intensity (*p* < 0.001) and reached an effect level close to the high intensity (0.50 ≤ effect size d < 0.8). It indicates that HR changes were largely influenced by the exercise intensity. This finding indicates that there is a significant difference in the magnitude of HR variation in runners at different intensities of exercise, and this finding is consistent with the common knowledge that there is a strong correlation between the magnitude of HR variation in runners and the intensity of exercise. In addition, a study by Szabo et al. provides evidence for the idea that music not only has an effect on fatigue perception during progressive exercise, but can still provide effects for a period of time at higher intensities, depending on the quality of intrinsic arousal [49]. The main reason is that the greater intensity of exercise causes a greater cardiac load, and the maintenance of cardiac output depends on an increase in HR; the fatigue of the body also increases, resulting in a higher HR immediately after running [50].

However, the main effect of music tempo was not significant (*p* > 0.05), and the level of effect size was small (0 < effect size d < 0.5), indicating that the magnitude of HR change during exercise was influenced by music tempo to a lesser extent. This finding is inconsistent with some current studies which indicate that the magnitude of HR variation during exercise is influenced by music tempo [51]. This finding is also inconsistent with the significant main effect of music tempo on the TFP, probably because the TFP is a subjective index of fatigue that reflects runners’ self-evaluation of their physical state and is significantly influenced by mental aspects. In contrast, under control conditions, the HR variation of the subjects mainly depends on subjects’ physical fatigue [52]. As some studies pointed out, the effect of music on fatigue perception would be reflected in the shift of runners’ attention to the fatigue state; that is, it would be shown first in the shift in perception of mental fatigue and then in the perception of physical fatigue [53,54]. However, the duration of this experiment was determined by the runners’ subjective fatigue, and the runners ended the run when their subjective fatigue reached the level of RPE = 15, which led to the intervention effect of music tempo on the runners’ physical fatigue perception not being fully demonstrated in this experiment. This further led to a nonsignificant interaction effect between music tempo and exercise intensity (*p* > 0.05, 0 < effect size d < 0.5). However, there are studies that have come to similar conclusions as the present study, such as Edworthy and Waring and Dyck et al., who also did not find a link between music tempo changes and HR changes [17,55]. HR and music tempo can be considered as interacting oscillatory systems that will start at the same period, but this alignment strategy only works at the beginning of the experiment.

### 4.3. Effect of Music Tempo on the Difference in MF at Different Exercise Intensities

The differences in MF for the RF and VM for different exercise regimens varied widely and did not show regular changes. The results show that the main effects of music tempo and exercise intensity on both MF_RF_ difference and MF_VM_ difference were not significant (*p* > 0.05) and that both had small effect sizes (0 < effect size d < 0.5). The interaction effects of music tempo and exercise intensity on MF_RF_ and MF_VM_ differences were also not significant (*p* > 0.05) and both effect sizes were small (0 < effect size d < 0.5). Meanwhile, the experimental results for the differences in MF were negative, which indicates decreased local muscle fatigue after running, which is inconsistent with the results of most previous studies that used the difference in MF as an indicator [56,57]. The reason may be due to the different distribution patterns of fast and slow muscle fibers in different individuals and muscles, which are genetically determined and almost impossible to change later by exercise [58]. In the post-experiment communication with the subjects, we learned that runners may use different force generation methods for different running conditions, which also indirectly influenced the experimental results. Meanwhile, the ANOVA results also show that the main effects of both exercise intensity and music tempo were not significant, indicating that the change in muscle fatigue before and after running was not significant. However, in the post-experiment questioning of the subjects, we found that the subjects all had some soreness and swelling in their lower limb muscles after finishing the running experiment. This phenomenon was not monitored during the experiment, and the sEMG signal changes for the two muscles before and after running were not regular; there were more negative differences in MF, which shows that the MF values in this experiment did not show a decreasing trend but an increasing trend. The reasons for this result in this study may be twofold. (1) The experimental test time was short, and the runners reached the specified value of subjective fatigue in a short period of time, during which the local muscle tissue continuously recruited fast muscle fibers to participate in the exercise so that the muscle firing frequency increased, and the neural excitability continued during the running process [59,60]. (2) The energy consumption during muscle contraction during running did not exceed the energy recovery during relaxation, and the lactic acid accumulation was not significant, so the driving strategy of the central nervous system for the muscles and the conduction speed of the muscle fibers were not affected [61]. Other scholars have also found different trends in MF frequency domain indicators during exercise [62]. Combining the above views to see the limitations of using the decrease in the MF frequency domain index pre- to post-exercise can determine the fatigue of local muscles during dynamic exercise such as running.

### 4.4. Pratical Applications

This study aimed to determine the effect of music tempo on runners’ fatigue perception at different exercise intensities and provided a physiological and psychological explanation of the role of music tempo in resisting fatigue perception by observing time of fatigue perception, heart rate changes and sEMG signal changes during the running experiment. The combined results of the three indicators revealed that the use of music with different tempos at different exercise intensities caused runners to exhibit different mental and physical performances. Specifically, runners’ TFP was influenced by the music tempo, and in addition, runners’ TFP was also influenced by the interaction between music tempo and exercise intensity. The change in HR of runners during running was mainly influenced by the exercise intensity. Whether it is low-intensity exercise or high-intensity exercise, listening to fast music may mitigate individuals’ perception of fatigue to some extent. This was also pointed out in the study by Centala et al. The effect of listening to fast music during exercise would be better than slow music and could regulate mental emotions and reduce the perception of fatigue during exercise [63].

In addition, this study also provides many practical implications. Running is an endurance sport, and runners will feel fatigue to different degrees during running, especially for many non-professional runners, who often do not persist in running due to insufficient exercise, limited physical strength and lack of perseverance. The results of this study can help runners to develop a more beneficial exercise program. For example, both low-intensity exercisers and high-intensity exercisers are advised to listen to fast music while exercising, which would help them maintain a better mental state, make the exercise process less tedious and better control the physiological state, including heart rate changes. This research could lead to better effects of music on perceived fatigue, allowing runners to have better mental and physical performance while running.

### 4.5. Limitations

There were some limitations to this study. First, the subject population was not subdivided, and none of the runners had undergone professional training. Second, we only examined male subjects. Future studies could use sex as an independent variable to discuss the effects of music tempo on subjects of different sexes and could also include people of all ages and from different occupational backgrounds to make the study results more generalizable. Third, the observation indexes used in this study were relatively limited, and more measurement techniques could be integrated in the future to more accurately assess the fatigue perception status of runners. Fourth, based on this study, future studies could refine the characteristics of the music, such as the cultural background of the music, or use music with lyrics to explore other methods of music intervention during exercise to promote the popularity of the research results.

## 5. Conclusions

In this paper, the effect of music tempo on runners’ subjective and objective fatigue perception at different exercise intensities was investigated through running experiments. The influence of music on runners’ perception of fatigue was explored, and the interaction between music tempo and exercise intensity was considered. The results of the study showed that (1) there were significant main effects and interaction effects of music tempo and exercise intensity on the TFP; (2) in terms of the difference in HR, the main effect of exercise intensity was significant, the main effect of music tempo was not significant and the interaction effect of music tempo and exercise intensity was not significant; and (3) in terms of the difference in the MF of sEMG, the main effect and interaction effect of music tempo and exercise intensity were not significant, and the results did not show regular changes. The combined results of the study indicate that fast music can effectively reduce the perception of fatigue of runners during running. The results of this study provide a scientific basis for ordinary runners to select evidence-based for running, help them effectively overcome their perception of fatigue during exercise and optimally modify their perception of fatigue with music.

## Figures and Tables

**Table 1:** Basic information of the subjects (M ± SD).

Number of Subjects	Age (Years)	Height (cm)	Weight (kg)	Resting Heart Rate (Beats/min)
18	23.95 ± 1.49	173.78 ± 1.54	64.39 ± 4.55	78.97 ± 8.15

**Table 2:** Details of music selection.

Music Tempo	Tracks	Duration/min	Beat/bpm
Slow music	Falcom Sound Team Jdk	4′23″	90
	Grass Harvest	3′14″	96
	The des Alizes	3′40″	100
	Harunouta	3′03″	90
	Sakurairo Contrail	2′28″	90
	Springtime Affair	2′49″	95
	Regrettably, You Know	2′04″	96
	A Tiny Sunshine	1′57″	99
	Sakura Residential Area	2′05″	97
Fast music	Cigarette Daydreams	3′12″	150
	Hero	3′34″	150
	Shanghai Alice Magic Orchestra	3′36″	152
	Toy War	1′55″	160
	Where to Jun	3′57″	160
	Dream Land Days	3′17″	155

**Table 3:** Borg’s scale for rating of perceived exertion.

Score	Subjective Exercise Intensity	Subjective Exercise Fatigue	Score	Subjective Exercise Intensity	Subjective Exercise Fatigue
6	No exertion at all	Not hard at all	14	-	
7	Extremely light	Extremely relaxed	15	Hard (heavy)	Tired
8	- ^1^		16	-	
9	Very light	Very relaxed	17	Very hard	Very tired
10	-		18	-	
11	Light	Relaxed	19	Extremely hard	Extremely tired
12	-		20	Maximal exertion	Trying one’s best
13	Somewhat hard	A little tired			

^1^-represents the fatigue status between the two levels.

**Table 4:** Examination of the effects of music tempo and exercise intensity.

Parameter	High			Low			Music Tempo		Exercise Intensity		Music Tempo × Exercise Intensity	
	Fast	Slow	None	Fast	Slow	None	*p*-Value	η^2^	*p*-Value	η^2^	*p*-Value	η^2^
TFP (min)	7.18 ± 2.36	6.23 ± 2.44	5.40 ± 1.94	12.68 ± 6.46	10.79 ± 4.86	9.06 ± 4.36	0.000	0.632	0.000	0.540	0.031	0.207
HR difference ^1^	69.56 ± 10.66	67.39 ± 11.14	71.11 ± 10.18	57.89 ± 10.30	61.83 ± 10.81	62.06 ± 8.95	0.077	0.140	0.000	0.796	0.075	0.141
MF_RF_ difference ^2^	−0.64 ± 4.98	−2.02 ± 2.95	0.53 ± 4.45	−0.86 ± 6.34	−0.42 ± 6.81	−0.96 ± 5.11	0.672	0.023	0.967	0.000	0.210	0.088
MF_VM_ difference ^2^	−0.75 ± 5.91	−0.89 ± 3.81	1.01 ± 4.2	0.38 ± 3.81	−1.08 ± 5.6	−0.79 ± 4.41	0.609	0.029	0.670	0.011	0.323	0.064

^1^ HR difference value is post-run HR−resting HR. ^2^ MF difference is MF before running−MF after running. RF: rectus femoris. VM: vastus medialis.

**Table 5:** Post hoc test of TFP under different experimental conditions.

Exercise Intensity	Pairwise Comparisons		
	No Music vs.	Slow Music vs.	Fast Music vs.
Low intensity	Slow music: *p* = 0.004	No music: *p* = 0.004	No music: *p* = 0.000
	Fast music: *p* = 0.000	Fast music: *p* = 0.000	Slow music: *p* = 0.000
High intensity	Slow music: *p* = 0.000	No music: *p* = 0.000	No music: *p* = 0.0000
	Fast music: *p* = 0.000	Fast music: *p* = 0.000	Slow music: *p* = 0.000

## Data Availability

Not applicable.

## Supplementary Materials

The following are available online at https://www.mdpi.com/article/10.3390/ijerph19073869/s1, File S1: Ethic reviews; File S2: Informed Consent.
