# Supplementary material for: The Effect of Music Tempo on Fatigue Perception at Different Exercise Intensities
Source: Int J Environ Res Public Health. 2022 Mar 24;19(7):3869. doi: 10.3390/ijerph19073869 (PMC8997730; doi:10.3390/ijerph19073869)
Supplement: Supplementary file 1 [file ijerph-19-03869-s001.zip › ijerph-1618544-supplementary/supplementary files/File S1-Ethics Review.pdf]

浙江工业大学工业设计研究院实验伦理审查表

Institute of Industrial Design of Zhejiang University of Technology

Experiment Ethics Review

项目编号：0903/2021

<table>
<tr><td>项目名称
(Project Name)</td><td>音乐节奏对不同强度跑步疲劳感知的影响研究
(The Effect of Music Tempo on Fatigue Perception at Different Exercise Intensities)</td></tr>
<tr><td>项目负责人
(Project leader)</td><td>吴剑锋 Jianfeng （签字）</td></tr>
<tr><td>所在部门
(Department)</td><td>浙江工业大学工业设计研究院
(Industrial Design& Research Institute, Zhejiang University of Technology)
浙江工业大学工业设计研究院</td></tr>
<tr><td>项目起止时间
(Time of start and stop)</td><td>2021.9.15-2021.10.15</td></tr>
<tr><td>伦理审查内容
(Content of ethical review)</td><td>研究内容：
本研究设计了以音乐节奏(快节奏、慢节奏、无音乐)和运动强度(高运动强度、低运动强度)为自变量的被试内双因素实验，以疲劳感知产生时间、心率信号变化、目标肌肉表面肌电(sEMG)信号的时频域指标变化为观测指标，邀请 13 名参与者完成了共 78 组跑步实验。
目标测试肌肉：结合前人的研究，发现股直肌(RF)、股内侧肌(VM)的肌电信号变化明显，并且能在动作变化时保持较强的稳定性。因此选定这两块肌肉作为本次实验的检测对象。
Research contents:
In this study, we designed a within-subject two-factor experiment with music tempo (fast rhythm, slow rhythm, no music) and exercise intensity (high exercise intensity, low exercise intensity) as independent variables, and invited 13 participants to complete a total of 78 sets of running experiments with fatigue perception generation time, heart rate signal changes, and changes in time-frequency domain indicators of surface electromyography (sEMG) signals of the target muscles as observations.
Target test muscles: Combined with previous studies, it was found that the electromyographic signals of rectus femoris (RF) and medial femoris (VM)muscles changed significantly and could maintain a strong stability during the movement changes. Therefore, these two muscles were selected as the test subjects for this experiment.

参与者：
13 名成年健康的，无肌肉、骨骼、呼吸系统或心血管等方面的疾病男性，身高 170-180cm，体重 60-75kg。
Subjects:
13 healthy adult males without musculoskeletal, respiratory or cardiovascular disease, height 170-180cm, weight 60-75kg.

相关影响：
本研究不会向被试者收取任何费用，也不能给被试者带来直接利益，但这个项目的研究将可能带来一些学术上的益处。本研究的结果可能会在学术期刊/书籍上发表，或者</td></tr>
</table>

	用于教学。被试者的名字及其他信息将不会在任何发表或教学的材料中出现，除非得到被试者的允许。如果被试者在本研究过程中受伤且受伤是研究程序的直接结果，我们将给被试者提供必要的治疗。<br>**Related influence**<br>No charges will be billed to you for this study.There is no other direct benefit of your participation in this experiment. However, research in this area may eventually be helpful for science.The results of this study may be published in an academic journal/book or used for teaching purposes. However, subjects' name or other identifiers will not be used in any publication or teaching materials without their specific permission.If subject is injured during the course of the study and as a direct result of this study, he could contact the investigator at the number provided. Subject will be offered the necessary care to treat that injury.	
审查专家组 (Expert group)	刘肖健(Xiaojian Liu)	教授(professor)
	黄薇(Wei Huang)	教授(professor)
	朱上上(Shangshang Zhu)	教授(professor)
审查意见	同意<br>Approved<br>审查专家代表签名: 刘肖健 Liu Xiangjian<br>Wei Huang<br>Shangshang Zhu<br>2021 年 9 月 15 日	
