# Supplementary material for: The Effect of Music Tempo on Fatigue Perception at Different Exercise Intensities
Source: Int J Environ Res Public Health. 2022 Mar 24;19(7):3869. doi: 10.3390/ijerph19073869 (PMC8997730; doi:10.3390/ijerph19073869)
Supplement: Supplementary file 1 [file ijerph-19-03869-s001.zip › ijerph-1618544-supplementary/supplementary files/File S2-Informed Consent.pdf]

知情同意书

INFORMED CONSENT

欢迎参加浙江工业大学工业设计研究院的研究！
参加本研究是自愿的，您可以随时退出而不会因此受到惩罚或遭受利益方面的损失。
本《知情同意书》会简要介绍该研究及其潜在的风险和不适，请认真阅读。

Welcome to participate in the research of the Industrial Design Research Institute of Zhejiang University of Technology!

Participation in this study is voluntary, and you can withdraw at any time without being punished or suffering loss of benefits.

This "Informed Consent" will briefly introduce the research and its potential risks and discomforts, please read it carefully.

1. 课题题目：音乐节奏对不同强度跑步疲劳感知的影响研究

1. Topic: Study on the Effect of Music Tempo on Fatigue Perception in Different Exercise Intensity

课题负责人信息 Information of Project Leader			
姓　名/ Name	吴剑锋 Jianfeng Wu	电　话/Telephone	18658127685
E-mail	Jianfw@126.com	传　真/Facsimile	/

2. 研究目的 Research purpose

明确不同运动强度下音乐节奏对跑步者疲劳感知的影响。

To clarify the effect of music tempo on runners' fatigue perception under different exercise intensities and music tempo.

3. 哪些人可以参加本实验 Who can participate in this experiment

无肌肉、骨骼、呼吸系统或心血管等方面的疾病的男性。

The men without musculoskeletal, respiratory or cardiovascular disease.

4. 哪些人不可以参加本实验 Who is not allowed to participate in this experiment

患有心肺疾病或肌肉功能损伤的人。

People with heart and lung disease or impaired muscle function.

5. 肌电记录技术简介 Introduction to EMG recording technology

肌电描记法（electromyography） 记录肌肉纤维活动引起的电位变化的方法。其结果成为肌电图。

Electromyography is a method of recording the potential changes caused by muscle fiber activity. The result is EMG.

6. 实验任务介绍 Introduction to experimental tasks

为了明确不同运动强度下音乐节奏对跑步者疲劳感知的影响，本研究拟展开跑步实验，通过在实验过程中采集跑步者跑步时的疲劳感知观测指标，分析和讨论跑步者在不同运动强度和音乐节奏条件下的疲劳感知的变化，以深入音乐节奏对疲劳感知干预作用的进一步理解，为帮助个体健身者在不同运动强度下选择音乐类型提供指导依据。

In order to clarify the effect of music tempo on runners' fatigue perception under different exercise intensities, this study intends to conduct a running experiment, and analyze and discuss the changes of runners' fatigue perception under different exercise intensities and music tempo conditions by collecting runners' fatigue perception observation indexes during the experiment, in order to further understand the intervention effect of music tempo on fatigue perception and provide guidance basis for helping individual fitness practitioners to choose music types under different exercise intensities.

7. 可能的风险和不适 Possible risks and discomforts

我们会使用酒精棉擦拭您腿部周围的部位并贴上电极，这可能会造成一定的不适。

We will use alcohol cotton to wipe the area around your leg and stick electrodes, which may cause certain discomfort.

8. 益处 Benefits

本研究对科学，特别是认知神经科学有潜在的贡献，但是对您个人无直接的益处。如果您是学生，参加本实验能够帮您更直观地了解压力分布与肌电这种研究方法。

This research has potential contributions to science, especially cognitive neuroscience, but has no direct benefit to you personally. If you are a student, participating in this experiment can help you understand the research methods of pressure distribution and electromyography more intuitively.

9. 报酬 Remuneration

50 CNY.

10. 实验结果的反馈 Feedback of experimental results

如果对您对本研究感兴趣，可以留下联系方式给主试。本项研究结束时，我们会向您反馈本研究的主要结果。请注意，我们提供的结果是所有参与者的平均，我们不会向您提供您个人的研究数据。

If you are interested in this research, you can leave your contact information to the examiner. At the end of this research, we will give you feedback on the main results of this research. Please note that the results we provide are the average of all participants, and we will not provide you with your personal research data.

11. 您的权利 Your rights

- 您有权利获知本研究的相关信息及可能的风险和不适。
- 您自愿参加本实验；您可以随时中止参与实验并获得与参与时间相应的报酬。
- 您的数据会被以匿名方式处理，并不被用于本研究以外的其他目的。
- 您的个人隐私会受到保护。您的名字或者其他身份信息不会在任何发表物或教学材料中出现，除非得到您的书面授权。
- You have the right to be informed about the relevant information and possible risks and discomforts of this study.
- You voluntarily participate in this experiment; you can stop participating in the experiment at any time and get rewards corresponding to the time of participation.
- Your data will be processed anonymously and will not be used for purposes other than this research.
- Your personal privacy will be protected. Your name or other identifying information will not appear in any publications or teaching materials unless you have your written authorization.

12. 您的义务 Your obligations

- 科学是严肃的，请遵从任务指导语和主试的要求，认真完成实验任务。请关闭手机。
- 如果不明白实验任务的要求，请及时告诉主试。
- 如果您有不适于参加该实验的清况（如癫痫），请告知主试。
- 您在参加实验前不可使用药物或饮酒。
- 请不要触碰、移动或调节实验设备。
- 在任务进行过程中，请尽可能避免身体动作，以便我们能够获取可靠的研究数据。您的配合程度将决定实验的成败。
- Science is serious, please follow the task instructions and the requirements of the examiner, and complete the experimental tasks carefully. Please turn off your phone.
- If you do not understand the requirements of the experimental task, please tell the test supervisor in time.
- If you have any clear conditions that are not suitable for participating in the experiment (such as epilepsy), please inform the examiner.
- You must not use drugs or drink alcohol before participating in the experiment.
- Do not touch, move or adjust the laboratory equipment.
  During the task, please avoid body movements as much as possible so that we can obtain reliable research data. Your level of cooperation will determine the success or failure of the experiment.

13.联系方式

本知情同意书您可以保留一份副本。如果您有其他与本研究相关的问题，可以与__张玲燕__联系。

You may keep a copy of this informed consent. If you have any other questions related to this study, please contact Lingyan Zhang.

电话/Telephone：15869118003

Email: lingyanzh@outlook.com

14. 被试声明 Statement of subjects

我确认已经被告知本研究的目的、过程、可能的风险和不适，以及潜在的益处。我关心的所有问题都已得到满意的回答。我已经详细阅读了本《被试知情同意书》，明白自己的权利和义务。本人明白可以随时退出本研究而不会因此受到惩罚或遭受利益方面的损失。本人同意在浙江工业大学工业设计研究院参加本研究。

I confirm that I have been informed of the purpose, process, possible risks and discomfort, and potential benefits of this study. All my concerns have been satisfactorily answered. I have read the informed consent form of the subjects in detail and understand my rights and obligations. I understand that I can withdraw from this study at any time without any punishment or loss of interest. I agree to participate in this study in the Institute of industrial design, Zhejiang University of technology.

被试签名：张浩楠　日期：2021.9.17

15. 主试声明 Statement of host

我已经向被试解释了研究的目的、研究的程序、潜在的危险和不适，以及她 / 他的权益。我已经尽最大可能回答了被试提出的与研究有关的问题。我声明将遵从肌电实验的标准作业程序。

I have explained to the subject the purpose of the study, the procedure of the study, the potential dangers and discomfort, and her / his rights and interests. I have tried my best to answer the questions related to the research. I declare that I will follow the standard operating procedures for the relevant experiments.

主试签名：江璐　日期：2021.9.17

NO. 2

知情同意书

INFORMED CONSENT

欢迎参加浙江工业大学工业设计研究院的研究！
参加本研究是自愿的，您可以随时退出而不会因此受到惩罚或遭受利益方面的损失。
本《知情同意书》会简要介绍该研究及其潜在的风险和不适，请认真阅读。

Welcome to participate in the research of the Industrial Design Research Institute of Zhejiang University of Technology!

Participation in this study is voluntary, and you can withdraw at any time without being punished or suffering loss of benefits.

This "Informed Consent" will briefly introduce the research and its potential risks and discomforts, please read it carefully.

1. 课题题目：音乐节奏对不同强度跑步疲劳感知的影响研究

1. Topic: Study on the Effect of Music Tempo on Fatigue Perception in Different Exercise Intensity

课题负责人信息 Information of Project Leader			
姓　名/ Name	吴剑锋 Jianfeng Wu	电　话/Telephone	18658127685
E-mail	Jianfw@126.com	传　真/Facsimile	/

2. 研究目的 Research purpose

明确不同运动强度下音乐节奏对跑步者疲劳感知的影响。

To clarify the effect of music tempo on runners' fatigue perception under different exercise intensities and music tempo.

3. 哪些人可以参加本实验 Who can participate in this experiment

无肌肉、骨骼、呼吸系统或心血管等方面的疾病的男性。

The men without musculoskeletal, respiratory or cardiovascular disease.

4. 哪些人不可以参加本实验 Who is not allowed to participate in this experiment

患有心肺疾病或肌肉功能损伤的人。

People with heart and lung disease or impaired muscle function.

5. 肌电记录技术简介 Introduction to EMG recording technology

肌电描记法（electromyography） 记录肌肉纤维活动引起的电位变化的方法。其结果成为肌电图。

Electromyography is a method of recording the potential changes caused by muscle fiber activity. The result is EMG.

6. 实验任务介绍 Introduction to experimental tasks

为了明确不同运动强度下音乐节奏对跑步者疲劳感知的影响，本研究拟展开跑步实验，通过在实验过程中采集跑步者跑步时的疲劳感知观测指标，分析和讨论跑步者在不同运动强度和音乐节奏条件下的疲劳感知的变化，以深入音乐节奏对疲劳感知干预作用的进一步理解，为帮助个体健身者在不同运动强度下选择音乐类型提供指导依据。

In order to clarify the effect of music tempo on runners' fatigue perception under different exercise intensities, this study intends to conduct a running experiment, and analyze and discuss the changes of runners' fatigue perception under different exercise intensities and music tempo conditions by collecting runners' fatigue perception observation indexes during the experiment, in order to further understand the intervention effect of music tempo on fatigue perception and provide guidance basis for helping individual fitness practitioners to choose music types under different exercise intensities.

7. 可能的风险和不适 Possible risks and discomforts

我们会使用酒精棉擦拭您腿部周围的部位并贴上电极，这可能会造成一定的不适。

We will use alcohol cotton to wipe the area around your leg and stick electrodes, which may cause certain discomfort.

8. 益处 Benefits

本研究对科学，特别是认知神经科学有潜在的贡献，但是对您个人无直接的益处。如果您是学生，参加本实验能够帮您更直观地了解压力分布与肌电这种研究方法。

This research has potential contributions to science, especially cognitive neuroscience, but has no direct benefit to you personally. If you are a student, participating in this experiment can help you understand the research methods of pressure distribution and electromyography more intuitively.

9. 报酬 Remuneration

50 CNY.

10. 实验结果的反馈 Feedback of experimental results

如果对您对本研究感兴趣，可以留下联系方式给主试。本项研究结束时，我们会向您反馈本研究的主要结果。请注意，我们提供的结果是所有参与者的平均，我们不会向您提供您个人的研究数据。

If you are interested in this research, you can leave your contact information to the examiner. At the end of this research, we will give you feedback on the main results of this research. Please note that the results we provide are the average of all participants, and we will not provide you with your personal research data.

11. 您的权利 Your rights

- 您有权利获知本研究的相关信息及可能的风险和不适。
- 您自愿参加本实验；您可以随时中止参与实验并获得与参与时间相应的报酬。
- 您的数据会被以匿名方式处理，并不被用于本研究以外的其他目的。
- 您的个人隐私会受到保护。您的名字或者其他身份信息不会在任何发表物或教学材料中出现，除非得到您的书面授权。
- You have the right to be informed about the relevant information and possible risks and discomforts of this study.
- You voluntarily participate in this experiment; you can stop participating in the experiment at any time and get rewards corresponding to the time of participation.
- Your data will be processed anonymously and will not be used for purposes other than this research.
- Your personal privacy will be protected. Your name or other identifying information will not appear in any publications or teaching materials unless you have your written authorization.

12. 您的义务 Your obligations

- 科学是严肃的，请遵从任务指导语和主试的要求，认真完成实验任务。请关闭手机。
- 如果不明白实验任务的要求，请及时告诉主试。
- 如果您有不适于参加该实验的清况（如癫痫），请告知主试。
- 您在参加实验前不可使用药物或饮酒。
- 请不要触碰、移动或调节实验设备。
- 在任务进行过程中，请尽可能避免身体动作，以便我们能够获取可靠的研究数据。您的配合程度将决定实验的成败。
- Science is serious, please follow the task instructions and the requirements of the examiner, and complete the experimental tasks carefully. Please turn off your phone.
- If you do not understand the requirements of the experimental task, please tell the test supervisor in time.
- If you have any clear conditions that are not suitable for participating in the experiment (such as epilepsy), please inform the examiner.
- You must not use drugs or drink alcohol before participating in the experiment.
- Do not touch, move or adjust the laboratory equipment.
  During the task, please avoid body movements as much as possible so that we can obtain reliable research data. Your level of cooperation will determine the success or failure of the experiment.

13.联系方式

本知情同意书您可以保留一份副本。如果您有其他与本研究相关的问题，可以与 张玲燕 联系。

You may keep a copy of this informed consent. If you have any other questions related to this study, please contact Lingyan Zhang.

电话/Telephone：15869118003
Email: lingyanzh@outlook.com

14. 被试声明 Statement of subjects

我确认已经被告知本研究的目的、过程、可能的风险和不适，以及潜在的益处。我关心的所有问题都已得到满意的回答。我已经详细阅读了本《被试知情同意书》，明白自己的权利和义务。本人明白可以随时退出本研究而不会因此受到惩罚或遭受利益方面的损失。本人同意在浙江工业大学工业设计研究院参加本研究。

I confirm that I have been informed of the purpose, process, possible risks and discomfort, and potential benefits of this study. All my concerns have been satisfactorily answered. I have read the informed consent form of the subjects in detail and understand my rights and obligations. I understand that I can withdraw from this study at any time without any punishment or loss of interest. I agree to participate in this study in the Institute of industrial design, Zhejiang University of technology.

被试签名：林[illegible] 日期：2021.9.17

15. 主试声明 Statement of host

我已经向被试解释了研究的目的、研究的程序、潜在的危险和不适，以及她 / 他的权益。我已经尽最大可能回答了被试提出的与研究有关的问题。我声明将遵从肌电实验的标准作业程序。

I have explained to the subject the purpose of the study, the procedure of the study, the potential dangers and discomfort, and her / his rights and interests. I have tried my best to answer the questions related to the research. I declare that I will follow the standard operating procedures for the relevant experiments.

主试签名：江[illegible] 日期：2021.9.17

NO. 3

知情同意书

INFORMED CONSENT

欢迎参加浙江工业大学工业设计研究院的研究！
参加本研究是自愿的，您可以随时退出而不会因此受到惩罚或遭受利益方面的损失。
本《知情同意书》会简要介绍该研究及其潜在的风险和不适，请认真阅读。

Welcome to participate in the research of the Industrial Design Research Institute of Zhejiang University of Technology!

Participation in this study is voluntary, and you can withdraw at any time without being punished or suffering loss of benefits.

This "Informed Consent" will briefly introduce the research and its potential risks and discomforts, please read it carefully.

1. 课题题目：音乐节奏对不同强度跑步疲劳感知的影响研究

1. Topic: Study on the Effect of Music Tempo on Fatigue Perception in Different Exercise Intensity

课题负责人信息 Information of Project Leader			
姓　名/ Name	吴剑锋 Jianfeng Wu	电　话/Telephone	18658127685
E-mail	Jianfw@126.com	传　真/Facsimile	/

2. 研究目的 Research purpose

明确不同运动强度下音乐节奏对跑步者疲劳感知的影响。

To clarify the effect of music tempo on runners' fatigue perception under different exercise intensities and music tempo.

3. 哪些人可以参加本实验 Who can participate in this experiment

无肌肉、骨骼、呼吸系统或心血管等方面的疾病的男性。

The men without musculoskeletal, respiratory or cardiovascular disease.

4. 哪些人不可以参加本实验 Who is not allowed to participate in this experiment

患有心肺疾病或肌肉功能损伤的人。

People with heart and lung disease or impaired muscle function.

5. 肌电记录技术简介 Introduction to EMG recording technology

肌电描记法（electromyography） 记录肌肉纤维活动引起的电位变化的方法。其结果成为肌电图。

Electromyography is a method of recording the potential changes caused by muscle fiber activity. The result is EMG.

6. 实验任务介绍 Introduction to experimental tasks

为了明确不同运动强度下音乐节奏对跑步者疲劳感知的影响，本研究拟展开跑步实验，通过在实验过程中采集跑步者跑步时的疲劳感知观测指标，分析和讨论跑步者在不同运动强度和音乐节奏条件下的疲劳感知的变化，以深入音乐节奏对疲劳感知干预作用的进一步理解，为帮助个体健身者在不同运动强度下选择音乐类型提供指导依据。

In order to clarify the effect of music tempo on runners' fatigue perception under different exercise intensities, this study intends to conduct a running experiment, and analyze and discuss the changes of runners' fatigue perception under different exercise intensities and music tempo conditions by collecting runners' fatigue perception observation indexes during the experiment, in order to further understand the intervention effect of music tempo on fatigue perception and provide guidance basis for helping individual fitness practitioners to choose music types under different exercise intensities.

7. 可能的风险和不适 Possible risks and discomforts

我们会使用酒精棉擦拭您腿部周围的部位并贴上电极，这可能会造成一定的不适。

We will use alcohol cotton to wipe the area around your leg and stick electrodes, which may cause certain discomfort.

8. 益处 Benefits

本研究对科学，特别是认知神经科学有潜在的贡献，但是对您个人无直接的益处。如果您是学生，参加本实验能够帮您更直观地了解压力分布与肌电这种研究方法。

This research has potential contributions to science, especially cognitive neuroscience, but has no direct benefit to you personally. If you are a student, participating in this experiment can help you understand the research methods of pressure distribution and electromyography more intuitively.

9. 报酬 Remuneration

50 CNY.

10. 实验结果的反馈 Feedback of experimental results

如果对您对本研究感兴趣，可以留下联系方式给主试。本项研究结束时，我们会向您反馈本研究的主要结果。请注意，我们提供的结果是所有参与者的平均，我们不会向您提供您个人的研究数据。

If you are interested in this research, you can leave your contact information to the examiner. At the end of this research, we will give you feedback on the main results of this research. Please note that the results we provide are the average of all participants, and we will not provide you with your personal research data.

11. 您的权利 Your rights

- 您有权利获知本研究的相关信息及可能的风险和不适。
- 您自愿参加本实验；您可以随时中止参与实验并获得与参与时间相应的报酬。
- 您的数据会被以匿名方式处理，并不被用于本研究以外的其他目的。
- 您的个人隐私会受到保护。您的名字或者其他身份信息不会在任何发表物或教学材料中出现，除非得到您的书面授权。
- You have the right to be informed about the relevant information and possible risks and discomforts of this study.
- You voluntarily participate in this experiment; you can stop participating in the experiment at any time and get rewards corresponding to the time of participation.
- Your data will be processed anonymously and will not be used for purposes other than this research.
- Your personal privacy will be protected. Your name or other identifying information will not appear in any publications or teaching materials unless you have your written authorization.

12. 您的义务 Your obligations

- 科学是严肃的，请遵从任务指导语和主试的要求，认真完成实验任务。请关闭手机。
- 如果不明白实验任务的要求，请及时告诉主试。
- 如果您有不适于参加该实验的清况（如癫痫），请告知主试。
- 您在参加实验前不可使用药物或饮酒。
- 请不要触碰、移动或调节实验设备。
- 在任务进行过程中，请尽可能避免身体动作，以便我们能够获取可靠的研究数据。您的配合程度将决定实验的成败。
- Science is serious, please follow the task instructions and the requirements of the examiner, and complete the experimental tasks carefully. Please turn off your phone.
- If you do not understand the requirements of the experimental task, please tell the test supervisor in time.
- If you have any clear conditions that are not suitable for participating in the experiment (such as epilepsy), please inform the examiner.
- You must not use drugs or drink alcohol before participating in the experiment.
- Do not touch, move or adjust the laboratory equipment.
  During the task, please avoid body movements as much as possible so that we can obtain reliable research data. Your level of cooperation will determine the success or failure of the experiment.

13.联系方式

本知情同意书您可以保留一份副本。如果您有其他与本研究相关的问题，可以与__张玲燕__联系。

You may keep a copy of this informed consent. If you have any other questions related to this study, please contact Lingyan Zhang.

电话/Telephone：15869118003

Email: lingyanzh@outlook.com

14. 被试声明 Statement of subjects

我确认已经被告知本研究的目的、过程、可能的风险和不适，以及潜在的益处。我关心的所有问题都已得到满意的回答。我已经详细阅读了本《被试知情同意书》，明白自己的权利和义务。本人明白可以随时退出本研究而不会因此受到惩罚或遭受利益方面的损失。本人同意在浙江工业大学工业设计研究院参加本研究。

I confirm that I have been informed of the purpose, process, possible risks and discomfort, and potential benefits of this study. All my concerns have been satisfactorily answered. I have read the informed consent form of the subjects in detail and understand my rights and obligations. I understand that I can withdraw from this study at any time without any punishment or loss of interest. I agree to participate in this study in the Institute of industrial design, Zhejiang University of technology.

被试签名：[illegible] 日期：2021.9.17

15. 主试声明 Statement of host

我已经向被试解释了研究的目的、研究的程序、潜在的危险和不适，以及她 / 他的权益。我已经尽最大可能回答了被试提出的与研究有关的问题。我声明将遵从肌电实验的标准作业程序。

I have explained to the subject the purpose of the study, the procedure of the study, the potential dangers and discomfort, and her / his rights and interests. I have tried my best to answer the questions related to the research. I declare that I will follow the standard operating procedures for the relevant experiments.

主试签名：[illegible] 日期：2021.9.17

NO. 4

知情同意书

INFORMED CONSENT

欢迎参加浙江工业大学工业设计研究院的研究！
参加本研究是自愿的，您可以随时退出而不会因此受到惩罚或遭受利益方面的损失。
本《知情同意书》会简要介绍该研究及其潜在的风险和不适，请认真阅读。

Welcome to participate in the research of the Industrial Design Research Institute of Zhejiang University of Technology!

Participation in this study is voluntary, and you can withdraw at any time without being punished or suffering loss of benefits.

This "Informed Consent" will briefly introduce the research and its potential risks and discomforts, please read it carefully.

1. 课题题目：音乐节奏对不同强度跑步疲劳感知的影响研究

1. Topic: Study on the Effect of Music Tempo on Fatigue Perception in Different Exercise Intensity

课题负责人信息 Information of Project Leader			
姓　名/ Name	吴剑锋 Jianfeng Wu	电　话/Telephone	18658127685
E-mail	Jianfw@126.com	传　真/Facsimile	/

2. 研究目的 Research purpose

明确不同运动强度下音乐节奏对跑步者疲劳感知的影响。

To clarify the effect of music tempo on runners' fatigue perception under different exercise intensities and music tempo.

3. 哪些人可以参加本实验 Who can participate in this experiment

无肌肉、骨骼、呼吸系统或心血管等方面的疾病的男性。

The men without musculoskeletal, respiratory or cardiovascular disease.

4. 哪些人不可以参加本实验 Who is not allowed to participate in this experiment

患有心肺疾病或肌肉功能损伤的人。

People with heart and lung disease or impaired muscle function.

5. 肌电记录技术简介 Introduction to EMG recording technology

肌电描记法（electromyography） 记录肌肉纤维活动引起的电位变化的方法。其结果成为肌电图。

Electromyography is a method of recording the potential changes caused by muscle fiber activity. The result is EMG.

6. 实验任务介绍 Introduction to experimental tasks

为了明确不同运动强度下音乐节奏对跑步者疲劳感知的影响，本研究拟展开跑步实验，通过在实验过程中采集跑步者跑步时的疲劳感知观测指标，分析和讨论跑步者在不同运动强度和音乐节奏条件下的疲劳感知的变化，以深入音乐节奏对疲劳感知干预作用的进一步理解，为帮助个体健身者在不同运动强度下选择音乐类型提供指导依据。

In order to clarify the effect of music tempo on runners' fatigue perception under different exercise intensities, this study intends to conduct a running experiment, and analyze and discuss the changes of runners' fatigue perception under different exercise intensities and music tempo conditions by collecting runners' fatigue perception observation indexes during the experiment, in order to further understand the intervention effect of music tempo on fatigue perception and provide guidance basis for helping individual fitness practitioners to choose music types under different exercise intensities.

7. 可能的风险和不适 Possible risks and discomforts

我们会使用酒精棉擦拭您腿部周围的部位并贴上电极，这可能会造成一定的不适。

We will use alcohol cotton to wipe the area around your leg and stick electrodes, which may cause certain discomfort.

8. 益处 Benefits

本研究对科学，特别是认知神经科学有潜在的贡献，但是对您个人无直接的益处。如果您是学生，参加本实验能够帮您更直观地了解压力分布与肌电这种研究方法。

This research has potential contributions to science, especially cognitive neuroscience, but has no direct benefit to you personally. If you are a student, participating in this experiment can help you understand the research methods of pressure distribution and electromyography more intuitively.

9. 报酬 Remuneration

50 CNY.

10. 实验结果的反馈 Feedback of experimental results

如果对您对本研究感兴趣，可以留下联系方式给主试。本项研究结束时，我们会向您反馈本研究的主要结果。请注意，我们提供的结果是所有参与者的平均，我们不会向您提供您个人的研究数据。

If you are interested in this research, you can leave your contact information to the examiner. At the end of this research, we will give you feedback on the main results of this research. Please note that the results we provide are the average of all participants, and we will not provide you with your personal research data.

11. 您的权利 Your rights

- 您有权利获知本研究的相关信息及可能的风险和不适。
- 您自愿参加本实验；您可以随时中止参与实验并获得与参与时间相应的报酬。
- 您的数据会被以匿名方式处理，并不被用于本研究以外的其他目的。
- 您的个人隐私会受到保护。您的名字或者其他身份信息不会在任何发表物或教学材料中出现，除非得到您的书面授权。
- You have the right to be informed about the relevant information and possible risks and discomforts of this study.
- You voluntarily participate in this experiment; you can stop participating in the experiment at any time and get rewards corresponding to the time of participation.
- Your data will be processed anonymously and will not be used for purposes other than this research.
- Your personal privacy will be protected. Your name or other identifying information will not appear in any publications or teaching materials unless you have your written authorization.

12. 您的义务 Your obligations

- 科学是严肃的，请遵从任务指导语和主试的要求，认真完成实验任务。请关闭手机。
- 如果不明白实验任务的要求，请及时告诉主试。
- 如果您有不适于参加该实验的清况（如癫痫），请告知主试。
- 您在参加实验前不可使用药物或饮酒。
- 请不要触碰、移动或调节实验设备。
- 在任务进行过程中，请尽可能避免身体动作，以便我们能够获取可靠的研究数据。您的配合程度将决定实验的成败。
- Science is serious, please follow the task instructions and the requirements of the examiner, and complete the experimental tasks carefully. Please turn off your phone.
- If you do not understand the requirements of the experimental task, please tell the test supervisor in time.
- If you have any clear conditions that are not suitable for participating in the experiment (such as epilepsy), please inform the examiner.
- You must not use drugs or drink alcohol before participating in the experiment.
- Do not touch, move or adjust the laboratory equipment.
  During the task, please avoid body movements as much as possible so that we can obtain reliable research data. Your level of cooperation will determine the success or failure of the experiment.

13.联系方式

本知情同意书您可以保留一份副本。如果您有其他与本研究相关的问题，可以与<u>张玲燕</u>联系。

You may keep a copy of this informed consent. If you have any other questions related to this study, please contact Lingyan Zhang.

电话/Telephone：15869118003
Email: lingyanzh@outlook.com

14. 被试声明 Statement of subjects

我确认已经被告知本研究的目的、过程、可能的风险和不适，以及潜在的益处。我关心的所有问题都已得到满意的回答。我已经详细阅读了本《被试知情同意书》，明白自己的权利和义务。本人明白可以随时退出本研究而不会因此受到惩罚或遭受利益方面的损失。本人同意在浙江工业大学工业设计研究院参加本研究。

I confirm that I have been informed of the purpose, process, possible risks and discomfort, and potential benefits of this study. All my concerns have been satisfactorily answered. I have read the informed consent form of the subjects in detail and understand my rights and obligations. I understand that I can withdraw from this study at any time without any punishment or loss of interest. I agree to participate in this study in the Institute of industrial design, Zhejiang University of technology.

被试签名：[illegible] 日期：2021.9.19

15. 主试声明 Statement of host

我已经向被试解释了研究的目的、研究的程序、潜在的危险和不适，以及她 / 他的权益。我已经尽最大可能回答了被试提出的与研究有关的问题。我声明将遵从肌电实验的标准作业程序。

I have explained to the subject the purpose of the study, the procedure of the study, the potential dangers and discomfort, and her / his rights and interests. I have tried my best to answer the questions related to the research. I declare that I will follow the standard operating procedures for the relevant experiments.

主试签名：[illegible] 日期：2021.9.19

NO. 5

知情同意书

INFORMED CONSENT

欢迎参加浙江工业大学工业设计研究院的研究！
参加本研究是自愿的，您可以随时退出而不会因此受到惩罚或遭受利益方面的损失。
本《知情同意书》会简要介绍该研究及其潜在的风险和不适，请认真阅读。

Welcome to participate in the research of the Industrial Design Research Institute of Zhejiang University of Technology!

Participation in this study is voluntary, and you can withdraw at any time without being punished or suffering loss of benefits.

This "Informed Consent" will briefly introduce the research and its potential risks and discomforts, please read it carefully.

1. 课题题目：音乐节奏对不同强度跑步疲劳感知的影响研究

1. Topic: Study on the Effect of Music Tempo on Fatigue Perception in Different Exercise Intensity

课题负责人信息 Information of Project Leader			
姓　名/ Name	吴剑锋 Jianfeng Wu	电　话/Telephone	18658127685
E-mail	Jianfw@126.com	传　真/Facsimile	/

2. 研究目的 Research purpose

明确不同运动强度下音乐节奏对跑步者疲劳感知的影响。

To clarify the effect of music tempo on runners' fatigue perception under different exercise intensities and music tempo.

3. 哪些人可以参加本实验 Who can participate in this experiment

无肌肉、骨骼、呼吸系统或心血管等方面的疾病的男性。

The men without musculoskeletal, respiratory or cardiovascular disease.

4. 哪些人不可以参加本实验 Who is not allowed to participate in this experiment

患有心肺疾病或肌肉功能损伤的人。

People with heart and lung disease or impaired muscle function.

5. 肌电记录技术简介 Introduction to EMG recording technology

肌电描记法（electromyography） 记录肌肉纤维活动引起的电位变化的方法。其结果成为肌电图。

Electromyography is a method of recording the potential changes caused by muscle fiber activity. The result is EMG.

6. 实验任务介绍 Introduction to experimental tasks

为了明确不同运动强度下音乐节奏对跑步者疲劳感知的影响，本研究拟展开跑步实验，通过在实验过程中采集跑步者跑步时的疲劳感知观测指标，分析和讨论跑步者在不同运动强度和音乐节奏条件下的疲劳感知的变化，以深入音乐节奏对疲劳感知干预作用的进一步理解，为帮助个体健身者在不同运动强度下选择音乐类型提供指导依据。

In order to clarify the effect of music tempo on runners' fatigue perception under different exercise intensities, this study intends to conduct a running experiment, and analyze and discuss the changes of runners' fatigue perception under different exercise intensities and music tempo conditions by collecting runners' fatigue perception observation indexes during the experiment, in order to further understand the intervention effect of music tempo on fatigue perception and provide guidance basis for helping individual fitness practitioners to choose music types under different exercise intensities.

7. 可能的风险和不适 Possible risks and discomforts

我们会使用酒精棉擦拭您腿部周围的部位并贴上电极，这可能会造成一定的不适。

We will use alcohol cotton to wipe the area around your leg and stick electrodes, which may cause certain discomfort.

8. 益处 Benefits

本研究对科学，特别是认知神经科学有潜在的贡献，但是对您个人无直接的益处。如果您是学生，参加本实验能够帮您更直观地了解压力分布与肌电这种研究方法。

This research has potential contributions to science, especially cognitive neuroscience, but has no direct benefit to you personally. If you are a student, participating in this experiment can help you understand the research methods of pressure distribution and electromyography more intuitively.

9. 报酬 Remuneration

50 CNY.

10. 实验结果的反馈 Feedback of experimental results

如果对您对本研究感兴趣，可以留下联系方式给主试。本项研究结束时，我们会向您反馈本研究的主要结果。请注意，我们提供的结果是所有参与者的平均，我们不会向您提供您个人的研究数据。

If you are interested in this research, you can leave your contact information to the examiner. At the end of this research, we will give you feedback on the main results of this research. Please note that the results we provide are the average of all participants, and we will not provide you with your personal research data.

11. 您的权利 Your rights

- 您有权利获知本研究的相关信息及可能的风险和不适。
- 您自愿参加本实验；您可以随时中止参与实验并获得与参与时间相应的报酬。
- 您的数据会被以匿名方式处理，并不被用于本研究以外的其他目的。
- 您的个人隐私会受到保护。您的名字或者其他身份信息不会在任何发表物或教学材料中出现，除非得到您的书面授权。
- You have the right to be informed about the relevant information and possible risks and discomforts of this study.
- You voluntarily participate in this experiment; you can stop participating in the experiment at any time and get rewards corresponding to the time of participation.
- Your data will be processed anonymously and will not be used for purposes other than this research.
- Your personal privacy will be protected. Your name or other identifying information will not appear in any publications or teaching materials unless you have your written authorization.

12. 您的义务 Your obligations

- 科学是严肃的，请遵从任务指导语和主试的要求，认真完成实验任务。请关闭手机。
- 如果不明白实验任务的要求，请及时告诉主试。
- 如果您有不适于参加该实验的清况（如癫痫），请告知主试。
- 您在参加实验前不可使用药物或饮酒。
- 请不要触碰、移动或调节实验设备。
- 在任务进行过程中，请尽可能避免身体动作，以便我们能够获取可靠的研究数据。您的配合程度将决定实验的成败。
- Science is serious, please follow the task instructions and the requirements of the examiner, and complete the experimental tasks carefully. Please turn off your phone.
- If you do not understand the requirements of the experimental task, please tell the test supervisor in time.
- If you have any clear conditions that are not suitable for participating in the experiment (such as epilepsy), please inform the examiner.
- You must not use drugs or drink alcohol before participating in the experiment.
- Do not touch, move or adjust the laboratory equipment.
  During the task, please avoid body movements as much as possible so that we can obtain reliable research data. Your level of cooperation will determine the success or failure of the experiment.

13.联系方式

本知情同意书您可以保留一份副本。如果您有其他与本研究相关的问题，可以与<u>张玲燕</u>联系。

You may keep a copy of this informed consent. If you have any other questions related to this study, please contact Lingyan Zhang.

电话/Telephone：15869118003

Email: lingyanzh@outlook.com

浙江工业大学工业设计研究院人体实验知情同意书

(Institute of Industrial Design of Zhejiang University of Technology Informed consent for human experiment)

14. 被试声明 Statement of subjects

我确认已经被告知本研究的目的、过程、可能的风险和不适，以及潜在的益处。我关心的所有问题都已得到满意的回答。我已经详细阅读了本《被试知情同意书》，明白自己的权利和义务。本人明白可以随时退出本研究而不会因此受到惩罚或遭受利益方面的损失。本人同意在浙江工业大学工业设计研究院参加本研究。

I confirm that I have been informed of the purpose, process, possible risks and discomfort, and potential benefits of this study. All my concerns have been satisfactorily answered. I have read the informed consent form of the subjects in detail and understand my rights and obligations. I understand that I can withdraw from this study at any time without any punishment or loss of interest. I agree to participate in this study in the Institute of industrial design, Zhejiang University of technology.

被试签名：[illegible] 日期：2021.9.19

15. 主试声明 Statement of host

我已经向被试解释了研究的目的、研究的程序、潜在的危险和不适，以及她 / 他的权益。我已经尽最大可能回答了被试提出的与研究有关的问题。我声明将遵从肌电实验的标准作业程序。

I have explained to the subject the purpose of the study, the procedure of the study, the potential dangers and discomfort, and her / his rights and interests. I have tried my best to answer the questions related to the research. I declare that I will follow the standard operating procedures for the relevant experiments.

主试签名：[illegible] 日期：2021.9.19

NO. 6

知情同意书

INFORMED CONSENT

欢迎参加浙江工业大学工业设计研究院的研究！
参加本研究是自愿的，您可以随时退出而不会因此受到惩罚或遭受利益方面的损失。
本《知情同意书》会简要介绍该研究及其潜在的风险和不适，请认真阅读。

Welcome to participate in the research of the Industrial Design Research Institute of Zhejiang University of Technology!

Participation in this study is voluntary, and you can withdraw at any time without being punished or suffering loss of benefits.

This "Informed Consent" will briefly introduce the research and its potential risks and discomforts, please read it carefully.

1. 课题题目：音乐节奏对不同强度跑步疲劳感知的影响研究

1. Topic: Study on the Effect of Music Tempo on Fatigue Perception in Different Exercise Intensity

课题负责人信息 Information of Project Leader			
姓　名/ Name	吴剑锋 Jianfeng Wu	电　话/Telephone	18658127685
E-mail	Jianfw@126.com	传　真/Facsimile	/

2. 研究目的 Research purpose

明确不同运动强度下音乐节奏对跑步者疲劳感知的影响。

To clarify the effect of music tempo on runners' fatigue perception under different exercise intensities and music tempo.

3. 哪些人可以参加本实验 Who can participate in this experiment

无肌肉、骨骼、呼吸系统或心血管等方面的疾病的男性。

The men without musculoskeletal, respiratory or cardiovascular disease.

4. 哪些人不可以参加本实验 Who is not allowed to participate in this experiment

患有心肺疾病或肌肉功能损伤的人。

People with heart and lung disease or impaired muscle function.

5.肌电记录技术简介 Introduction to EMG recording technology

肌电描记法（electromyography） 记录肌肉纤维活动引起的电位变化的方法。其结果成为肌电图。

Electromyography is a method of recording the potential changes caused by muscle fiber activity. The result is EMG.

6.实验任务介绍 Introduction to experimental tasks

为了明确不同运动强度下音乐节奏对跑步者疲劳感知的影响，本研究拟展开跑步实验，通过在实验过程中采集跑步者跑步时的疲劳感知观测指标，分析和讨论跑步者在不同运动强度和音乐节奏条件下的疲劳感知的变化，以深入音乐节奏对疲劳感知干预作用的进一步理解，为帮助个体健身者在不同运动强度下选择音乐类型提供指导依据。

In order to clarify the effect of music tempo on runners' fatigue perception under different exercise intensities, this study intends to conduct a running experiment, and analyze and discuss the changes of runners' fatigue perception under different exercise intensities and music tempo conditions by collecting runners' fatigue perception observation indexes during the experiment, in order to further understand the intervention effect of music tempo on fatigue perception and provide guidance basis for helping individual fitness practitioners to choose music types under different exercise intensities.

7.可能的风险和不适 Possible risks and discomforts

我们会使用酒精棉擦拭您腿部周围的部位并贴上电极，这可能会造成一定的不适。

We will use alcohol cotton to wipe the area around your leg and stick electrodes, which may cause certain discomfort.

8.益处 Benefits

本研究对科学，特别是认知神经科学有潜在的贡献，但是对您个人无直接的益处。如果您是学生，参加本实验能够帮您更直观地了解压力分布与肌电这种研究方法。

This research has potential contributions to science, especially cognitive neuroscience, but has no direct benefit to you personally. If you are a student, participating in this experiment can help you understand the research methods of pressure distribution and electromyography more intuitively.

9.报酬 Remuneration

50 CNY.

10.实验结果的反馈 Feedback of experimental results

如果对您对本研究感兴趣，可以留下联系方式给主试。本项研究结束时，我们会向您反馈本研究的主要结果。请注意，我们提供的结果是所有参与者的平均，我们不会向您提供您个人的研究数据。

If you are interested in this research, you can leave your contact information to the examiner. At the end of this research, we will give you feedback on the main results of this research. Please note that the results we provide are the average of all participants, and we will not provide you with your personal research data.

11. 您的权利 Your rights

- 您有权利获知本研究的相关信息及可能的风险和不适。
- 您自愿参加本实验；您可以随时中止参与实验并获得与参与时间相应的报酬。
- 您的数据会被以匿名方式处理，并不被用于本研究以外的其他目的。
- 您的个人隐私会受到保护。您的名字或者其他身份信息不会在任何发表物或教学材料中出现，除非得到您的书面授权。
- You have the right to be informed about the relevant information and possible risks and discomforts of this study.
- You voluntarily participate in this experiment; you can stop participating in the experiment at any time and get rewards corresponding to the time of participation.
- Your data will be processed anonymously and will not be used for purposes other than this research.
- Your personal privacy will be protected. Your name or other identifying information will not appear in any publications or teaching materials unless you have your written authorization.

12. 您的义务 Your obligations

- 科学是严肃的，请遵从任务指导语和主试的要求，认真完成实验任务。请关闭手机。
- 如果不明白实验任务的要求，请及时告诉主试。
- 如果您有不适于参加该实验的清况（如癫痫），请告知主试。
- 您在参加实验前不可使用药物或饮酒。
- 请不要触碰、移动或调节实验设备。
- 在任务进行过程中，请尽可能避免身体动作，以便我们能够获取可靠的研究数据。您的配合程度将决定实验的成败。
- Science is serious, please follow the task instructions and the requirements of the examiner, and complete the experimental tasks carefully. Please turn off your phone.
- If you do not understand the requirements of the experimental task, please tell the test supervisor in time.
- If you have any clear conditions that are not suitable for participating in the experiment (such as epilepsy), please inform the examiner.
- You must not use drugs or drink alcohol before participating in the experiment.
- Do not touch, move or adjust the laboratory equipment.
  During the task, please avoid body movements as much as possible so that we can obtain reliable research data. Your level of cooperation will determine the success or failure of the experiment.

13.联系方式

本知情同意书您可以保留一份副本。如果您有其他与本研究相关的问题，可以与 张玲燕 联系。

You may keep a copy of this informed consent. If you have any other questions related to this study, please contact Lingyan Zhang.

电话/Telephone：15869118003

Email: lingyanzh@outlook.com

14. 被试声明 Statement of subjects

我确认已经被告知本研究的目的、过程、可能的风险和不适，以及潜在的益处。我关心的所有问题都已得到满意的回答。我已经详细阅读了本《被试知情同意书》，明白自己的权利和义务。本人明白可以随时退出本研究而不会因此受到惩罚或遭受利益方面的损失。本人同意在浙江工业大学工业设计研究院参加本研究。

I confirm that I have been informed of the purpose, process, possible risks and discomfort, and potential benefits of this study. All my concerns have been satisfactorily answered. I have read the informed consent form of the subjects in detail and understand my rights and obligations. I understand that I can withdraw from this study at any time without any punishment or loss of interest. I agree to participate in this study in the Institute of industrial design, Zhejiang University of technology.

被试签名：徐涵杰 日期：2021.9.23

15. 主试声明 Statement of host

我已经向被试解释了研究的目的、研究的程序、潜在的危险和不适，以及她 / 他的权益。我已经尽最大可能回答了被试提出的与研究有关的问题。我声明将遵从肌电实验的标准作业程序。

I have explained to the subject the purpose of the study, the procedure of the study, the potential dangers and discomfort, and her / his rights and interests. I have tried my best to answer the questions related to the research. I declare that I will follow the standard operating procedures for the relevant experiments.

主试签名：[illegible] 日期：2021.9.23

知情同意书

INFORMED CONSENT

欢迎参加浙江工业大学工业设计研究院的研究！
参加本研究是自愿的，您可以随时退出而不会因此受到惩罚或遭受利益方面的损失。
本《知情同意书》会简要介绍该研究及其潜在的风险和不适，请认真阅读。

Welcome to participate in the research of the Industrial Design Research Institute of Zhejiang University of Technology!

Participation in this study is voluntary, and you can withdraw at any time without being punished or suffering loss of benefits.

This "Informed Consent" will briefly introduce the research and its potential risks and discomforts, please read it carefully.

1. 课题题目：音乐节奏对不同强度跑步疲劳感知的影响研究

1. Topic: Study on the Effect of Music Tempo on Fatigue Perception in Different Exercise Intensity

课题负责人信息 Information of Project Leader			
姓 名/ Name	吴剑锋 Jianfeng Wu	电 话/Telephone	18658127685
E-mail	Jianfw@126.com	传 真/Facsimile	/

2. 研究目的 Research purpose

明确不同运动强度下音乐节奏对跑步者疲劳感知的影响。

To clarify the effect of music tempo on runners' fatigue perception under different exercise intensities and music tempo.

3. 哪些人可以参加本实验 Who can participate in this experiment

无肌肉、骨骼、呼吸系统或心血管等方面的疾病的男性。

The men without musculoskeletal, respiratory or cardiovascular disease.

4. 哪些人不可以参加本实验 Who is not allowed to participate in this experiment

患有心肺疾病或肌肉功能损伤的人。

People with heart and lung disease or impaired muscle function.

5. 肌电记录技术简介 Introduction to EMG recording technology

肌电描记法（electromyography） 记录肌肉纤维活动引起的电位变化的方法。其结果成为肌电图。

Electromyography is a method of recording the potential changes caused by muscle fiber activity. The result is EMG.

6. 实验任务介绍 Introduction to experimental tasks

为了明确不同运动强度下音乐节奏对跑步者疲劳感知的影响，本研究拟展开跑步实验，通过在实验过程中采集跑步者跑步时的疲劳感知观测指标，分析和讨论跑步者在不同运动强度和音乐节奏条件下的疲劳感知的变化，以深入音乐节奏对疲劳感知干预作用的进一步理解，为帮助个体健身者在不同运动强度下选择音乐类型提供指导依据。

In order to clarify the effect of music tempo on runners' fatigue perception under different exercise intensities, this study intends to conduct a running experiment, and analyze and discuss the changes of runners' fatigue perception under different exercise intensities and music tempo conditions by collecting runners' fatigue perception observation indexes during the experiment, in order to further understand the intervention effect of music tempo on fatigue perception and provide guidance basis for helping individual fitness practitioners to choose music types under different exercise intensities.

7. 可能的风险和不适 Possible risks and discomforts

我们会使用酒精棉擦拭您腿部周围的部位并贴上电极，这可能会造成一定的不适。

We will use alcohol cotton to wipe the area around your leg and stick electrodes, which may cause certain discomfort.

8. 益处 Benefits

本研究对科学，特别是认知神经科学有潜在的贡献，但是对您个人无直接的益处。如果您是学生，参加本实验能够帮您更直观地了解压力分布与肌电这种研究方法。

This research has potential contributions to science, especially cognitive neuroscience, but has no direct benefit to you personally. If you are a student, participating in this experiment can help you understand the research methods of pressure distribution and electromyography more intuitively.

9. 报酬 Remuneration

50 CNY.

10. 实验结果的反馈 Feedback of experimental results

如果对您对本研究感兴趣，可以留下联系方式给主试。本项研究结束时，我们会向您反馈本研究的主要结果。请注意，我们提供的结果是所有参与者的平均，我们不会向您提供您个人的研究数据。

If you are interested in this research, you can leave your contact information to the examiner. At the end of this research, we will give you feedback on the main results of this research. Please note that the results we provide are the average of all participants, and we will not provide you with your personal research data.

11. 您的权利 Your rights

- 您有权利获知本研究的相关信息及可能的风险和不适。
- 您自愿参加本实验；您可以随时中止参与实验并获得与参与时间相应的报酬。
- 您的数据会被以匿名方式处理，并不被用于本研究以外的其他目的。
- 您的个人隐私会受到保护。您的名字或者其他身份信息不会在任何发表物或教学材料中出现，除非得到您的书面授权。
- You have the right to be informed about the relevant information and possible risks and discomforts of this study.
- You voluntarily participate in this experiment; you can stop participating in the experiment at any time and get rewards corresponding to the time of participation.
- Your data will be processed anonymously and will not be used for purposes other than this research.
- Your personal privacy will be protected. Your name or other identifying information will not appear in any publications or teaching materials unless you have your written authorization.

12. 您的义务 Your obligations

- 科学是严肃的，请遵从任务指导语和主试的要求，认真完成实验任务。请关闭手机。
- 如果不明白实验任务的要求，请及时告诉主试。
- 如果您有不适于参加该实验的清况（如癫痫），请告知主试。
- 您在参加实验前不可使用药物或饮酒。
- 请不要触碰、移动或调节实验设备。
- 在任务进行过程中，请尽可能避免身体动作，以便我们能够获取可靠的研究数据。您的配合程度将决定实验的成败。
- Science is serious, please follow the task instructions and the requirements of the examiner, and complete the experimental tasks carefully. Please turn off your phone.
- If you do not understand the requirements of the experimental task, please tell the test supervisor in time.
- If you have any clear conditions that are not suitable for participating in the experiment (such as epilepsy), please inform the examiner.
- You must not use drugs or drink alcohol before participating in the experiment.
- Do not touch, move or adjust the laboratory equipment.
  During the task, please avoid body movements as much as possible so that we can obtain reliable research data. Your level of cooperation will determine the success or failure of the experiment.

13.联系方式

本知情同意书您可以保留一份副本。如果您有其他与本研究相关的问题，可以与__张玲燕__联系。

You may keep a copy of this informed consent. If you have any other questions related to this study, please contact Lingyan Zhang.

电话/Telephone：15869118003

Email: lingyanzh@outlook.com

14. 被试声明 Statement of subjects

我确认已经被告知本研究的目的、过程、可能的风险和不适，以及潜在的益处。我关心的所有问题都已得到满意的回答。我已经详细阅读了本《被试知情同意书》，明白自己的权利和义务。本人明白可以随时退出本研究而不会因此受到惩罚或遭受利益方面的损失。本人同意在浙江工业大学工业设计研究院参加本研究。

I confirm that I have been informed of the purpose, process, possible risks and discomfort, and potential benefits of this study. All my concerns have been satisfactorily answered. I have read the informed consent form of the subjects in detail and understand my rights and obligations. I understand that I can withdraw from this study at any time without any punishment or loss of interest. I agree to participate in this study in the Institute of industrial design, Zhejiang University of technology.

被试签名：李鑫泽 日期：2021.9.23

15. 主试声明 Statement of host

我已经向被试解释了研究的目的、研究的程序、潜在的危险和不适，以及她 / 他的权益。我已经尽最大可能回答了被试提出的与研究有关的问题。我声明将遵从肌电实验的标准作业程序。

I have explained to the subject the purpose of the study, the procedure of the study, the potential dangers and discomfort, and her / his rights and interests. I have tried my best to answer the questions related to the research. I declare that I will follow the standard operating procedures for the relevant experiments.

主试签名：[illegible] 日期：2021.9.23

知情同意书

INFORMED CONSENT

欢迎参加浙江工业大学工业设计研究院的研究！
参加本研究是自愿的，您可以随时退出而不会因此受到惩罚或遭受利益方面的损失。
本《知情同意书》会简要介绍该研究及其潜在的风险和不适，请认真阅读。

Welcome to participate in the research of the Industrial Design Research Institute of Zhejiang University of Technology!

Participation in this study is voluntary, and you can withdraw at any time without being punished or suffering loss of benefits.

This "Informed Consent" will briefly introduce the research and its potential risks and discomforts, please read it carefully.

1. 课题题目：音乐节奏对不同强度跑步疲劳感知的影响研究

1. Topic: Study on the Effect of Music Tempo on Fatigue Perception in Different Exercise Intensity

课题负责人信息 Information of Project Leader			
姓　名/ Name	吴剑锋 Jianfeng Wu	电　话/Telephone	18658127685
E-mail	Jianfw@126.com	传　真/Facsimile	/

2. 研究目的 Research purpose

明确不同运动强度下音乐节奏对跑步者疲劳感知的影响。

To clarify the effect of music tempo on runners' fatigue perception under different exercise intensities and music tempo.

3. 哪些人可以参加本实验 Who can participate in this experiment

无肌肉、骨骼、呼吸系统或心血管等方面的疾病的男性。

The men without musculoskeletal, respiratory or cardiovascular disease.

4. 哪些人不可以参加本实验 Who is not allowed to participate in this experiment

患有心肺疾病或肌肉功能损伤的人。

People with heart and lung disease or impaired muscle function.

5. 肌电记录技术简介 Introduction to EMG recording technology

肌电描记法（electromyography） 记录肌肉纤维活动引起的电位变化的方法。其结果成为肌电图。

Electromyography is a method of recording the potential changes caused by muscle fiber activity. The result is EMG.

6. 实验任务介绍 Introduction to experimental tasks

为了明确不同运动强度下音乐节奏对跑步者疲劳感知的影响，本研究拟展开跑步实验，通过在实验过程中采集跑步者跑步时的疲劳感知观测指标，分析和讨论跑步者在不同运动强度和音乐节奏条件下的疲劳感知的变化，以深入音乐节奏对疲劳感知干预作用的进一步理解，为帮助个体健身者在不同运动强度下选择音乐类型提供指导依据。

In order to clarify the effect of music tempo on runners' fatigue perception under different exercise intensities, this study intends to conduct a running experiment, and analyze and discuss the changes of runners' fatigue perception under different exercise intensities and music tempo conditions by collecting runners' fatigue perception observation indexes during the experiment, in order to further understand the intervention effect of music tempo on fatigue perception and provide guidance basis for helping individual fitness practitioners to choose music types under different exercise intensities.

7. 可能的风险和不适 Possible risks and discomforts

我们会使用酒精棉擦拭您腿部周围的部位并贴上电极，这可能会造成一定的不适。

We will use alcohol cotton to wipe the area around your leg and stick electrodes, which may cause certain discomfort.

8. 益处 Benefits

本研究对科学，特别是认知神经科学有潜在的贡献，但是对您个人无直接的益处。如果您是学生，参加本实验能够帮您更直观地了解压力分布与肌电这种研究方法。

This research has potential contributions to science, especially cognitive neuroscience, but has no direct benefit to you personally. If you are a student, participating in this experiment can help you understand the research methods of pressure distribution and electromyography more intuitively.

9. 报酬 Remuneration

50 CNY.

10. 实验结果的反馈 Feedback of experimental results

如果对您对本研究感兴趣，可以留下联系方式给主试。本项研究结束时，我们会向您反馈本研究的主要结果。请注意，我们提供的结果是所有参与者的平均，我们不会向您提供您个人的研究数据。

If you are interested in this research, you can leave your contact information to the examiner. At the end of this research, we will give you feedback on the main results of this research. Please note that the results we provide are the average of all participants, and we will not provide you with your personal research data.

11. 您的权利 Your rights

- 您有权利获知本研究的相关信息及可能的风险和不适。
- 您自愿参加本实验；您可以随时中止参与实验并获得与参与时间相应的报酬。
- 您的数据会被以匿名方式处理，并不被用于本研究以外的其他目的。
- 您的个人隐私会受到保护。您的名字或者其他身份信息不会在任何发表物或教学材料中出现，除非得到您的书面授权。
- You have the right to be informed about the relevant information and possible risks and discomforts of this study.
- You voluntarily participate in this experiment; you can stop participating in the experiment at any time and get rewards corresponding to the time of participation.
- Your data will be processed anonymously and will not be used for purposes other than this research.
- Your personal privacy will be protected. Your name or other identifying information will not appear in any publications or teaching materials unless you have your written authorization.

12. 您的义务 Your obligations

- 科学是严肃的，请遵从任务指导语和主试的要求，认真完成实验任务。请关闭手机。
- 如果不明白实验任务的要求，请及时告诉主试。
- 如果您有不适于参加该实验的清况（如癫痫），请告知主试。
- 您在参加实验前不可使用药物或饮酒。
- 请不要触碰、移动或调节实验设备。
- 在任务进行过程中，请尽可能避免身体动作，以便我们能够获取可靠的研究数据。您的配合程度将决定实验的成败。
- Science is serious, please follow the task instructions and the requirements of the examiner, and complete the experimental tasks carefully. Please turn off your phone.
- If you do not understand the requirements of the experimental task, please tell the test supervisor in time.
- If you have any clear conditions that are not suitable for participating in the experiment (such as epilepsy), please inform the examiner.
- You must not use drugs or drink alcohol before participating in the experiment.
- Do not touch, move or adjust the laboratory equipment.

  During the task, please avoid body movements as much as possible so that we can obtain reliable research data. Your level of cooperation will determine the success or failure of the experiment.

13.联系方式

本知情同意书您可以保留一份副本。如果您有其他与本研究相关的问题，可以与<u>张玲燕</u>联系。

You may keep a copy of this informed consent. If you have any other questions related to this study, please contact Lingyan Zhang.

电话/Telephone：15869118003

Email: lingyanzh@outlook.com

14. 被试声明 Statement of subjects

我确认已经被告知本研究的目的、过程、可能的风险和不适，以及潜在的益处。我关心的所有问题都已得到满意的回答。我已经详细阅读了本《被试知情同意书》，明白自己的权利和义务。本人明白可以随时退出本研究而不会因此受到惩罚或遭受利益方面的损失。本人同意在浙江工业大学工业设计研究院参加本研究。

I confirm that I have been informed of the purpose, process, possible risks and discomfort, and potential benefits of this study. All my concerns have been satisfactorily answered. I have read the informed consent form of the subjects in detail and understand my rights and obligations. I understand that I can withdraw from this study at any time without any punishment or loss of interest. I agree to participate in this study in the Institute of industrial design, Zhejiang University of technology.

被试签名：[illegible] 日期：2021.9.24

15. 主试声明 Statement of host

我已经向被试解释了研究的目的、研究的程序、潜在的危险和不适，以及她 / 他的权益。我已经尽最大可能回答了被试提出的与研究有关的问题。我声明将遵从肌电实验的标准作业程序。

I have explained to the subject the purpose of the study, the procedure of the study, the potential dangers and discomfort, and her / his rights and interests. I have tried my best to answer the questions related to the research. I declare that I will follow the standard operating procedures for the relevant experiments.

主试签名：[illegible] 日期：2021.9.24

NO. 9

知情同意书

INFORMED CONSENT

欢迎参加浙江工业大学工业设计研究院的研究！
参加本研究是自愿的，您可以随时退出而不会因此受到惩罚或遭受利益方面的损失。
本《知情同意书》会简要介绍该研究及其潜在的风险和不适，请认真阅读。

Welcome to participate in the research of the Industrial Design Research Institute of Zhejiang University of Technology!

Participation in this study is voluntary, and you can withdraw at any time without being punished or suffering loss of benefits.

This "Informed Consent" will briefly introduce the research and its potential risks and discomforts, please read it carefully.

1. 课题题目：音乐节奏对不同强度跑步疲劳感知的影响研究

1. Topic: Study on the Effect of Music Tempo on Fatigue Perception in Different Exercise Intensity

课题负责人信息 Information of Project Leader			
姓　名/ Name	吴剑锋 Jianfeng Wu	电　话/Telephone	18658127685
E-mail	Jianfw@126.com	传　真/Facsimile	/

2. 研究目的 Research purpose

明确不同运动强度下音乐节奏对跑步者疲劳感知的影响。

To clarify the effect of music tempo on runners' fatigue perception under different exercise intensities and music tempo.

3. 哪些人可以参加本实验 Who can participate in this experiment

无肌肉、骨骼、呼吸系统或心血管等方面的疾病的男性。

The men without musculoskeletal, respiratory or cardiovascular disease.

4. 哪些人不可以参加本实验 Who is not allowed to participate in this experiment

患有心肺疾病或肌肉功能损伤的人。

People with heart and lung disease or impaired muscle function.

5. 肌电记录技术简介 Introduction to EMG recording technology

肌电描记法（electromyography） 记录肌肉纤维活动引起的电位变化的方法。其结果成为肌电图。

Electromyography is a method of recording the potential changes caused by muscle fiber activity. The result is EMG.

6. 实验任务介绍 Introduction to experimental tasks

为了明确不同运动强度下音乐节奏对跑步者疲劳感知的影响，本研究拟展开跑步实验，通过在实验过程中采集跑步者跑步时的疲劳感知观测指标，分析和讨论跑步者在不同运动强度和音乐节奏条件下的疲劳感知的变化，以深入音乐节奏对疲劳感知干预作用的进一步理解，为帮助个体健身者在不同运动强度下选择音乐类型提供指导依据。

In order to clarify the effect of music tempo on runners' fatigue perception under different exercise intensities, this study intends to conduct a running experiment, and analyze and discuss the changes of runners' fatigue perception under different exercise intensities and music tempo conditions by collecting runners' fatigue perception observation indexes during the experiment, in order to further understand the intervention effect of music tempo on fatigue perception and provide guidance basis for helping individual fitness practitioners to choose music types under different exercise intensities.

7. 可能的风险和不适 Possible risks and discomforts

我们会使用酒精棉擦拭您腿部周围的部位并贴上电极，这可能会造成一定的不适。

We will use alcohol cotton to wipe the area around your leg and stick electrodes, which may cause certain discomfort.

8. 益处 Benefits

本研究对科学，特别是认知神经科学有潜在的贡献，但是对您个人无直接的益处。如果您是学生，参加本实验能够帮您更直观地了解压力分布与肌电这种研究方法。

This research has potential contributions to science, especially cognitive neuroscience, but has no direct benefit to you personally. If you are a student, participating in this experiment can help you understand the research methods of pressure distribution and electromyography more intuitively.

9. 报酬 Remuneration

50 CNY.

10. 实验结果的反馈 Feedback of experimental results

如果对您对本研究感兴趣，可以留下联系方式给主试。本项研究结束时，我们会向您反馈本研究的主要结果。请注意，我们提供的结果是所有参与者的平均，我们不会向您提供您个人的研究数据。

If you are interested in this research, you can leave your contact information to the examiner. At the end of this research, we will give you feedback on the main results of this research. Please note that the results we provide are the average of all participants, and we will not provide you with your personal research data.

11. 您的权利 Your rights

- 您有权利获知本研究的相关信息及可能的风险和不适。
- 您自愿参加本实验；您可以随时中止参与实验并获得与参与时间相应的报酬。
- 您的数据会被以匿名方式处理，并不被用于本研究以外的其他目的。
- 您的个人隐私会受到保护。您的名字或者其他身份信息不会在任何发表物或教学材料中出现，除非得到您的书面授权。
- You have the right to be informed about the relevant information and possible risks and discomforts of this study.
- You voluntarily participate in this experiment; you can stop participating in the experiment at any time and get rewards corresponding to the time of participation.
- Your data will be processed anonymously and will not be used for purposes other than this research.
- Your personal privacy will be protected. Your name or other identifying information will not appear in any publications or teaching materials unless you have your written authorization.

12. 您的义务 Your obligations

- 科学是严肃的，请遵从任务指导语和主试的要求，认真完成实验任务。请关闭手机。
- 如果不明白实验任务的要求，请及时告诉主试。
- 如果您有不适于参加该实验的清况（如癫痫），请告知主试。
- 您在参加实验前不可使用药物或饮酒。
- 请不要触碰、移动或调节实验设备。
- 在任务进行过程中，请尽可能避免身体动作，以便我们能够获取可靠的研究数据。您的配合程度将决定实验的成败。
- Science is serious, please follow the task instructions and the requirements of the examiner, and complete the experimental tasks carefully. Please turn off your phone.
- If you do not understand the requirements of the experimental task, please tell the test supervisor in time.
- If you have any clear conditions that are not suitable for participating in the experiment (such as epilepsy), please inform the examiner.
- You must not use drugs or drink alcohol before participating in the experiment.
- Do not touch, move or adjust the laboratory equipment.
  During the task, please avoid body movements as much as possible so that we can obtain reliable research data. Your level of cooperation will determine the success or failure of the experiment.

13.联系方式

本知情同意书您可以保留一份副本。如果您有其他与本研究相关的问题，可以与<u>张玲燕</u>联系。

You may keep a copy of this informed consent. If you have any other questions related to this study, please contact Lingyan Zhang.

电话/Telephone：15869118003
Email: lingyanzh@outlook.com

14. 被试声明 Statement of subjects

我确认已经被告知本研究的目的、过程、可能的风险和不适，以及潜在的益处。我关心的所有问题都已得到满意的回答。我已经详细阅读了本《被试知情同意书》，明白自己的权利和义务。本人明白可以随时退出本研究而不会因此受到惩罚或遭受利益方面的损失。本人同意在浙江工业大学工业设计研究院参加本研究。

I confirm that I have been informed of the purpose, process, possible risks and discomfort, and potential benefits of this study. All my concerns have been satisfactorily answered. I have read the informed consent form of the subjects in detail and understand my rights and obligations. I understand that I can withdraw from this study at any time without any punishment or loss of interest. I agree to participate in this study in the Institute of industrial design, Zhejiang University of technology.

被试签名：翁苏赞 日期：2021.9.28

15. 主试声明 Statement of host

我已经向被试解释了研究的目的、研究的程序、潜在的危险和不适，以及她 / 他的权益。我已经尽最大可能回答了被试提出的与研究有关的问题。我声明将遵从肌电实验的标准作业程序。

I have explained to the subject the purpose of the study, the procedure of the study, the potential dangers and discomfort, and her / his rights and interests. I have tried my best to answer the questions related to the research. I declare that I will follow the standard operating procedures for the relevant experiments.

主试签名：[illegible] 日期：2021.9.28

NO. 10

知情同意书

INFORMED CONSENT

欢迎参加浙江工业大学工业设计研究院的研究！

参加本研究是自愿的，您可以随时退出而不会因此受到惩罚或遭受利益方面的损失。

本《知情同意书》会简要介绍该研究及其潜在的风险和不适，请认真阅读。

Welcome to participate in the research of the Industrial Design Research Institute of Zhejiang University of Technology!

Participation in this study is voluntary, and you can withdraw at any time without being punished or suffering loss of benefits.

This "Informed Consent" will briefly introduce the research and its potential risks and discomforts, please read it carefully.

1. 课题题目：音乐节奏对不同强度跑步疲劳感知的影响研究

1. Topic: Study on the Effect of Music Tempo on Fatigue Perception in Different Exercise Intensity

课题负责人信息 Information of Project Leader			
姓　名/ Name	吴剑锋 Jianfeng Wu	电　话/Telephone	18658127685
E-mail	Jianfw@126.com	传　真/Facsimile	/

2. 研究目的 Research purpose

明确不同运动强度下音乐节奏对跑步者疲劳感知的影响。

To clarify the effect of music tempo on runners' fatigue perception under different exercise intensities and music tempo.

3. 哪些人可以参加本实验 Who can participate in this experiment

无肌肉、骨骼、呼吸系统或心血管等方面的疾病的男性。

The men without musculoskeletal, respiratory or cardiovascular disease.

4. 哪些人不可以参加本实验 Who is not allowed to participate in this experiment

患有心肺疾病或肌肉功能损伤的人。

People with heart and lung disease or impaired muscle function.

5. 肌电记录技术简介 Introduction to EMG recording technology

肌电描记法（electromyography） 记录肌肉纤维活动引起的电位变化的方法。其结果成为肌电图。

Electromyography is a method of recording the potential changes caused by muscle fiber activity. The result is EMG.

6. 实验任务介绍 Introduction to experimental tasks

为了明确不同运动强度下音乐节奏对跑步者疲劳感知的影响，本研究拟展开跑步实验，通过在实验过程中采集跑步者跑步时的疲劳感知观测指标，分析和讨论跑步者在不同运动强度和音乐节奏条件下的疲劳感知的变化，以深入音乐节奏对疲劳感知干预作用的进一步理解，为帮助个体健身者在不同运动强度下选择音乐类型提供指导依据。

In order to clarify the effect of music tempo on runners' fatigue perception under different exercise intensities, this study intends to conduct a running experiment, and analyze and discuss the changes of runners' fatigue perception under different exercise intensities and music tempo conditions by collecting runners' fatigue perception observation indexes during the experiment, in order to further understand the intervention effect of music tempo on fatigue perception and provide guidance basis for helping individual fitness practitioners to choose music types under different exercise intensities.

7. 可能的风险和不适 Possible risks and discomforts

我们会使用酒精棉擦拭您腿部周围的部位并贴上电极，这可能会造成一定的不适。

We will use alcohol cotton to wipe the area around your leg and stick electrodes, which may cause certain discomfort.

8. 益处 Benefits

本研究对科学，特别是认知神经科学有潜在的贡献，但是对您个人无直接的益处。如果您是学生，参加本实验能够帮您更直观地了解压力分布与肌电这种研究方法。

This research has potential contributions to science, especially cognitive neuroscience, but has no direct benefit to you personally. If you are a student, participating in this experiment can help you understand the research methods of pressure distribution and electromyography more intuitively.

9. 报酬 Remuneration

50 CNY.

10. 实验结果的反馈 Feedback of experimental results

如果对您对本研究感兴趣，可以留下联系方式给主试。本项研究结束时，我们会向您反馈本研究的主要结果。请注意，我们提供的结果是所有参与者的平均，我们不会向您提供您个人的研究数据。

If you are interested in this research, you can leave your contact information to the examiner. At the end of this research, we will give you feedback on the main results of this research. Please note that the results we provide are the average of all participants, and we will not provide you with your personal research data.

11. 您的权利 Your rights

- 您有权利获知本研究的相关信息及可能的风险和不适。
- 您自愿参加本实验；您可以随时中止参与实验并获得与参与时间相应的报酬。
- 您的数据会被以匿名方式处理，并不被用于本研究以外的其他目的。
- 您的个人隐私会受到保护。您的名字或者其他身份信息不会在任何发表物或教学材料中出现，除非得到您的书面授权。
- You have the right to be informed about the relevant information and possible risks and discomforts of this study.
- You voluntarily participate in this experiment; you can stop participating in the experiment at any time and get rewards corresponding to the time of participation.
- Your data will be processed anonymously and will not be used for purposes other than this research.
- Your personal privacy will be protected. Your name or other identifying information will not appear in any publications or teaching materials unless you have your written authorization.

12. 您的义务 Your obligations

- 科学是严肃的，请遵从任务指导语和主试的要求，认真完成实验任务。请关闭手机。
- 如果不明白实验任务的要求，请及时告诉主试。
- 如果您有不适于参加该实验的清况（如癫痫），请告知主试。
- 您在参加实验前不可使用药物或饮酒。
- 请不要触碰、移动或调节实验设备。
- 在任务进行过程中，请尽可能避免身体动作，以便我们能够获取可靠的研究数据。您的配合程度将决定实验的成败。
- Science is serious, please follow the task instructions and the requirements of the examiner, and complete the experimental tasks carefully. Please turn off your phone.
- If you do not understand the requirements of the experimental task, please tell the test supervisor in time.
- If you have any clear conditions that are not suitable for participating in the experiment (such as epilepsy), please inform the examiner.
- You must not use drugs or drink alcohol before participating in the experiment.
- Do not touch, move or adjust the laboratory equipment.
  During the task, please avoid body movements as much as possible so that we can obtain reliable research data. Your level of cooperation will determine the success or failure of the experiment.

13.联系方式

本知情同意书您可以保留一份副本。如果您有其他与本研究相关的问题，可以与<u>张玲燕</u>联系。

You may keep a copy of this informed consent. If you have any other questions related to this study, please contact Lingyan Zhang.

电话/Telephone：15869118003

Email: lingyanzh@outlook.com

14. 被试声明 Statement of subjects

我确认已经被告知本研究的目的、过程、可能的风险和不适，以及潜在的益处。我关心的所有问题都已得到满意的回答。我已经详细阅读了本《被试知情同意书》，明白自己的权利和义务。本人明白可以随时退出本研究而不会因此受到惩罚或遭受利益方面的损失。本人同意在浙江工业大学工业设计研究院参加本研究。

I confirm that I have been informed of the purpose, process, possible risks and discomfort, and potential benefits of this study. All my concerns have been satisfactorily answered. I have read the informed consent form of the subjects in detail and understand my rights and obligations. I understand that I can withdraw from this study at any time without any punishment or loss of interest. I agree to participate in this study in the Institute of industrial design, Zhejiang University of technology.

被试签名：许鹏程 日期：2021.10.8

15. 主试声明 Statement of host

我已经向被试解释了研究的目的、研究的程序、潜在的危险和不适，以及她 / 他的权益。我已经尽最大可能回答了被试提出的与研究有关的问题。我声明将遵从肌电实验的标准作业程序。

I have explained to the subject the purpose of the study, the procedure of the study, the potential dangers and discomfort, and her / his rights and interests. I have tried my best to answer the questions related to the research. I declare that I will follow the standard operating procedures for the relevant experiments.

主试签名：[illegible] 日期：2021.10.8

NO. 11

知情同意书

INFORMED CONSENT

欢迎参加浙江工业大学工业设计研究院的研究！
参加本研究是自愿的，您可以随时退出而不会因此受到惩罚或遭受利益方面的损失。
本《知情同意书》会简要介绍该研究及其潜在的风险和不适，请认真阅读。

Welcome to participate in the research of the Industrial Design Research Institute of Zhejiang University of Technology!

Participation in this study is voluntary, and you can withdraw at any time without being punished or suffering loss of benefits.

This "Informed Consent" will briefly introduce the research and its potential risks and discomforts, please read it carefully.

1. 课题题目：音乐节奏对不同强度跑步疲劳感知的影响研究

1. Topic: Study on the Effect of Music Tempo on Fatigue Perception in Different Exercise Intensity

课题负责人信息 Information of Project Leader			
姓　名/ Name	吴剑锋 Jianfeng Wu	电　话/Telephone	18658127685
E-mail	Jianfw@126.com	传　真/Facsimile	/

2. 研究目的 Research purpose

明确不同运动强度下音乐节奏对跑步者疲劳感知的影响。

To clarify the effect of music tempo on runners' fatigue perception under different exercise intensities and music tempo.

3. 哪些人可以参加本实验 Who can participate in this experiment

无肌肉、骨骼、呼吸系统或心血管等方面的疾病的男性。

The men without musculoskeletal, respiratory or cardiovascular disease.

4. 哪些人不可以参加本实验 Who is not allowed to participate in this experiment

患有心肺疾病或肌肉功能损伤的人。

People with heart and lung disease or impaired muscle function.

5. 肌电记录技术简介 Introduction to EMG recording technology

肌电描记法（electromyography） 记录肌肉纤维活动引起的电位变化的方法。其结果成为肌电图。

Electromyography is a method of recording the potential changes caused by muscle fiber activity. The result is EMG.

6. 实验任务介绍 Introduction to experimental tasks

为了明确不同运动强度下音乐节奏对跑步者疲劳感知的影响，本研究拟展开跑步实验，通过在实验过程中采集跑步者跑步时的疲劳感知观测指标，分析和讨论跑步者在不同运动强度和音乐节奏条件下的疲劳感知的变化，以深入音乐节奏对疲劳感知干预作用的进一步理解，为帮助个体健身者在不同运动强度下选择音乐类型提供指导依据。

In order to clarify the effect of music tempo on runners' fatigue perception under different exercise intensities, this study intends to conduct a running experiment, and analyze and discuss the changes of runners' fatigue perception under different exercise intensities and music tempo conditions by collecting runners' fatigue perception observation indexes during the experiment, in order to further understand the intervention effect of music tempo on fatigue perception and provide guidance basis for helping individual fitness practitioners to choose music types under different exercise intensities.

7. 可能的风险和不适 Possible risks and discomforts

我们会使用酒精棉擦拭您腿部周围的部位并贴上电极，这可能会造成一定的不适。

We will use alcohol cotton to wipe the area around your leg and stick electrodes, which may cause certain discomfort.

8. 益处 Benefits

本研究对科学，特别是认知神经科学有潜在的贡献，但是对您个人无直接的益处。如果您是学生，参加本实验能够帮您更直观地了解压力分布与肌电这种研究方法。

This research has potential contributions to science, especially cognitive neuroscience, but has no direct benefit to you personally. If you are a student, participating in this experiment can help you understand the research methods of pressure distribution and electromyography more intuitively.

9. 报酬 Remuneration

50 CNY.

10. 实验结果的反馈 Feedback of experimental results

如果对您对本研究感兴趣，可以留下联系方式给主试。本项研究结束时，我们会向您反馈本研究的主要结果。请注意，我们提供的结果是所有参与者的平均，我们不会向您提供您个人的研究数据。

If you are interested in this research, you can leave your contact information to the examiner. At the end of this research, we will give you feedback on the main results of this research. Please note that the results we provide are the average of all participants, and we will not provide you with your personal research data.

11. 您的权利 Your rights

- 您有权利获知本研究的相关信息及可能的风险和不适。
- 您自愿参加本实验；您可以随时中止参与实验并获得与参与时间相应的报酬。
- 您的数据会被以匿名方式处理，并不被用于本研究以外的其他目的。
- 您的个人隐私会受到保护。您的名字或者其他身份信息不会在任何发表物或教学材料中出现，除非得到您的书面授权。
- You have the right to be informed about the relevant information and possible risks and discomforts of this study.
- You voluntarily participate in this experiment; you can stop participating in the experiment at any time and get rewards corresponding to the time of participation.
- Your data will be processed anonymously and will not be used for purposes other than this research.
- Your personal privacy will be protected. Your name or other identifying information will not appear in any publications or teaching materials unless you have your written authorization.

12. 您的义务 Your obligations

- 科学是严肃的，请遵从任务指导语和主试的要求，认真完成实验任务。请关闭手机。
- 如果不明白实验任务的要求，请及时告诉主试。
- 如果您有不适于参加该实验的清况（如癫痫），请告知主试。
- 您在参加实验前不可使用药物或饮酒。
- 请不要触碰、移动或调节实验设备。
- 在任务进行过程中，请尽可能避免身体动作，以便我们能够获取可靠的研究数据。您的配合程度将决定实验的成败。
- Science is serious, please follow the task instructions and the requirements of the examiner, and complete the experimental tasks carefully. Please turn off your phone.
- If you do not understand the requirements of the experimental task, please tell the test supervisor in time.
- If you have any clear conditions that are not suitable for participating in the experiment (such as epilepsy), please inform the examiner.
- You must not use drugs or drink alcohol before participating in the experiment.
- Do not touch, move or adjust the laboratory equipment.
  During the task, please avoid body movements as much as possible so that we can obtain reliable research data. Your level of cooperation will determine the success or failure of the experiment.

13.联系方式

本知情同意书您可以保留一份副本。如果您有其他与本研究相关的问题，可以与<u>张玲燕</u>联系。

You may keep a copy of this informed consent. If you have any other questions related to this study, please contact Lingyan Zhang.

电话/Telephone：15869118003

Email: lingyanzh@outlook.com

14. 被试声明 Statement of subjects

我确认已经被告知本研究的目的、过程、可能的风险和不适，以及潜在的益处。我关心的所有问题都已得到满意的回答。我已经详细阅读了本《被试知情同意书》，明白自己的权利和义务。本人明白可以随时退出本研究而不会因此受到惩罚或遭受利益方面的损失。本人同意在浙江工业大学工业设计研究院参加本研究。

I confirm that I have been informed of the purpose, process, possible risks and discomfort, and potential benefits of this study. All my concerns have been satisfactorily answered. I have read the informed consent form of the subjects in detail and understand my rights and obligations. I understand that I can withdraw from this study at any time without any punishment or loss of interest. I agree to participate in this study in the Institute of industrial design, Zhejiang University of technology.

被试签名：[illegible] 日期：2021.10.8

15. 主试声明 Statement of host

我已经向被试解释了研究的目的、研究的程序、潜在的危险和不适，以及她 / 他的权益。我已经尽最大可能回答了被试提出的与研究有关的问题。我声明将遵从肌电实验的标准作业程序。

I have explained to the subject the purpose of the study, the procedure of the study, the potential dangers and discomfort, and her / his rights and interests. I have tried my best to answer the questions related to the research. I declare that I will follow the standard operating procedures for the relevant experiments.

主试签名：[illegible] 日期：2021.10.8

知情同意书

INFORMED CONSENT

欢迎参加浙江工业大学工业设计研究院的研究！
参加本研究是自愿的，您可以随时退出而不会因此受到惩罚或遭受利益方面的损失。
本《知情同意书》会简要介绍该研究及其潜在的风险和不适，请认真阅读。

Welcome to participate in the research of the Industrial Design Research Institute of Zhejiang University of Technology!

Participation in this study is voluntary, and you can withdraw at any time without being punished or suffering loss of benefits.

This "Informed Consent" will briefly introduce the research and its potential risks and discomforts, please read it carefully.

1. 课题题目：音乐节奏对不同强度跑步疲劳感知的影响研究

1. Topic: Study on the Effect of Music Tempo on Fatigue Perception in Different Exercise Intensity

课题负责人信息 Information of Project Leader			
姓　名/ Name	吴剑锋 Jianfeng Wu	电　话/Telephone	18658127685
E-mail	Jianfw@126.com	传　真/Facsimile	/

2. 研究目的 Research purpose

明确不同运动强度下音乐节奏对跑步者疲劳感知的影响。

To clarify the effect of music tempo on runners' fatigue perception under different exercise intensities and music tempo.

3. 哪些人可以参加本实验 Who can participate in this experiment

无肌肉、骨骼、呼吸系统或心血管等方面的疾病的男性。

The men without musculoskeletal, respiratory or cardiovascular disease.

4. 哪些人不可以参加本实验 Who is not allowed to participate in this experiment

患有心肺疾病或肌肉功能损伤的人。

People with heart and lung disease or impaired muscle function.

5. 肌电记录技术简介 Introduction to EMG recording technology

肌电描记法（electromyography） 记录肌肉纤维活动引起的电位变化的方法。其结果成为肌电图。

Electromyography is a method of recording the potential changes caused by muscle fiber activity. The result is EMG.

6. 实验任务介绍 Introduction to experimental tasks

为了明确不同运动强度下音乐节奏对跑步者疲劳感知的影响，本研究拟展开跑步实验，通过在实验过程中采集跑步者跑步时的疲劳感知观测指标，分析和讨论跑步者在不同运动强度和音乐节奏条件下的疲劳感知的变化，以深入音乐节奏对疲劳感知干预作用的进一步理解，为帮助个体健身者在不同运动强度下选择音乐类型提供指导依据。

In order to clarify the effect of music tempo on runners' fatigue perception under different exercise intensities, this study intends to conduct a running experiment, and analyze and discuss the changes of runners' fatigue perception under different exercise intensities and music tempo conditions by collecting runners' fatigue perception observation indexes during the experiment, in order to further understand the intervention effect of music tempo on fatigue perception and provide guidance basis for helping individual fitness practitioners to choose music types under different exercise intensities.

7. 可能的风险和不适 Possible risks and discomforts

我们会使用酒精棉擦拭您腿部周围的部位并贴上电极，这可能会造成一定的不适。

We will use alcohol cotton to wipe the area around your leg and stick electrodes, which may cause certain discomfort.

8. 益处 Benefits

本研究对科学，特别是认知神经科学有潜在的贡献，但是对您个人无直接的益处。如果您是学生，参加本实验能够帮您更直观地了解压力分布与肌电这种研究方法。

This research has potential contributions to science, especially cognitive neuroscience, but has no direct benefit to you personally. If you are a student, participating in this experiment can help you understand the research methods of pressure distribution and electromyography more intuitively.

9. 报酬 Remuneration

50 CNY.

10. 实验结果的反馈 Feedback of experimental results

如果对您对本研究感兴趣，可以留下联系方式给主试。本项研究结束时，我们会向您反馈本研究的主要结果。请注意，我们提供的结果是所有参与者的平均，我们不会向您提供您个人的研究数据。

If you are interested in this research, you can leave your contact information to the examiner. At the end of this research, we will give you feedback on the main results of this research. Please note that the results we provide are the average of all participants, and we will not provide you with your personal research data.

11. 您的权利 Your rights

- 您有权利获知本研究的相关信息及可能的风险和不适。
- 您自愿参加本实验；您可以随时中止参与实验并获得与参与时间相应的报酬。
- 您的数据会被以匿名方式处理，并不被用于本研究以外的其他目的。
- 您的个人隐私会受到保护。您的名字或者其他身份信息不会在任何发表物或教学材料中出现，除非得到您的书面授权。
- You have the right to be informed about the relevant information and possible risks and discomforts of this study.
- You voluntarily participate in this experiment; you can stop participating in the experiment at any time and get rewards corresponding to the time of participation.
- Your data will be processed anonymously and will not be used for purposes other than this research.
- Your personal privacy will be protected. Your name or other identifying information will not appear in any publications or teaching materials unless you have your written authorization.

12. 您的义务 Your obligations

- 科学是严肃的，请遵从任务指导语和主试的要求，认真完成实验任务。请关闭手机。
- 如果不明白实验任务的要求，请及时告诉主试。
- 如果您有不适于参加该实验的清况（如癫痫），请告知主试。
- 您在参加实验前不可使用药物或饮酒。
- 请不要触碰、移动或调节实验设备。
- 在任务进行过程中，请尽可能避免身体动作，以便我们能够获取可靠的研究数据。您的配合程度将决定实验的成败。
- Science is serious, please follow the task instructions and the requirements of the examiner, and complete the experimental tasks carefully. Please turn off your phone.
- If you do not understand the requirements of the experimental task, please tell the test supervisor in time.
- If you have any clear conditions that are not suitable for participating in the experiment (such as epilepsy), please inform the examiner.
- You must not use drugs or drink alcohol before participating in the experiment.
- Do not touch, move or adjust the laboratory equipment.
  During the task, please avoid body movements as much as possible so that we can obtain reliable research data. Your level of cooperation will determine the success or failure of the experiment.

13.联系方式

本知情同意书您可以保留一份副本。如果您有其他与本研究相关的问题，可以与 张玲燕 联系。

You may keep a copy of this informed consent. If you have any other questions related to this study, please contact Lingyan Zhang.

电话/Telephone：15869118003

Email: lingyanzh@outlook.com

14. 被试声明 Statement of subjects

我确认已经被告知本研究的目的、过程、可能的风险和不适，以及潜在的益处。我关心的所有问题都已得到满意的回答。我已经详细阅读了本《被试知情同意书》，明白自己的权利和义务。本人明白可以随时退出本研究而不会因此受到惩罚或遭受利益方面的损失。本人同意在浙江工业大学工业设计研究院参加本研究。

I confirm that I have been informed of the purpose, process, possible risks and discomfort, and potential benefits of this study. All my concerns have been satisfactorily answered. I have read the informed consent form of the subjects in detail and understand my rights and obligations. I understand that I can withdraw from this study at any time without any punishment or loss of interest. I agree to participate in this study in the Institute of industrial design, Zhejiang University of technology.

被试签名：[illegible] 日期：2021.10.10

15. 主试声明 Statement of host

我已经向被试解释了研究的目的、研究的程序、潜在的危险和不适，以及她 / 他的权益。我已经尽最大可能回答了被试提出的与研究有关的问题。我声明将遵从肌电实验的标准作业程序。

I have explained to the subject the purpose of the study, the procedure of the study, the potential dangers and discomfort, and her / his rights and interests. I have tried my best to answer the questions related to the research. I declare that I will follow the standard operating procedures for the relevant experiments.

主试签名：[illegible] 日期：2021.10.10

知情同意书

INFORMED CONSENT

欢迎参加浙江工业大学工业设计研究院的研究！
参加本研究是自愿的，您可以随时退出而不会因此受到惩罚或遭受利益方面的损失。
本《知情同意书》会简要介绍该研究及其潜在的风险和不适，请认真阅读。

Welcome to participate in the research of the Industrial Design Research Institute of Zhejiang University of Technology!

Participation in this study is voluntary, and you can withdraw at any time without being punished or suffering loss of benefits.

This "Informed Consent" will briefly introduce the research and its potential risks and discomforts, please read it carefully.

1. 课题题目：音乐节奏对不同强度跑步疲劳感知的影响研究

1. Topic: Study on the Effect of Music Tempo on Fatigue Perception in Different Exercise Intensity

课题负责人信息 Information of Project Leader			
姓　名/ Name	吴剑锋 Jianfeng Wu	电　话/Telephone	18658127685
E-mail	Jianfw@126.com	传　真/Facsimile	/

2. 研究目的 Research purpose

明确不同运动强度下音乐节奏对跑步者疲劳感知的影响。

To clarify the effect of music tempo on runners' fatigue perception under different exercise intensities and music tempo.

3. 哪些人可以参加本实验 Who can participate in this experiment

无肌肉、骨骼、呼吸系统或心血管等方面的疾病的男性。

The men without musculoskeletal, respiratory or cardiovascular disease.

4. 哪些人不可以参加本实验 Who is not allowed to participate in this experiment

患有心肺疾病或肌肉功能损伤的人。

People with heart and lung disease or impaired muscle function.

5.肌电记录技术简介 Introduction to EMG recording technology

肌电描记法（electromyography） 记录肌肉纤维活动引起的电位变化的方法。其结果成为肌电图。

Electromyography is a method of recording the potential changes caused by muscle fiber activity. The result is EMG.

6.实验任务介绍 Introduction to experimental tasks

为了明确不同运动强度下音乐节奏对跑步者疲劳感知的影响，本研究拟展开跑步实验，通过在实验过程中采集跑步者跑步时的疲劳感知观测指标，分析和讨论跑步者在不同运动强度和音乐节奏条件下的疲劳感知的变化，以深入音乐节奏对疲劳感知干预作用的进一步理解，为帮助个体健身者在不同运动强度下选择音乐类型提供指导依据。

In order to clarify the effect of music tempo on runners' fatigue perception under different exercise intensities, this study intends to conduct a running experiment, and analyze and discuss the changes of runners' fatigue perception under different exercise intensities and music tempo conditions by collecting runners' fatigue perception observation indexes during the experiment, in order to further understand the intervention effect of music tempo on fatigue perception and provide guidance basis for helping individual fitness practitioners to choose music types under different exercise intensities.

7.可能的风险和不适 Possible risks and discomforts

我们会使用酒精棉擦拭您腿部周围的部位并贴上电极，这可能会造成一定的不适。

We will use alcohol cotton to wipe the area around your leg and stick electrodes, which may cause certain discomfort.

8.益处 Benefits

本研究对科学，特别是认知神经科学有潜在的贡献，但是对您个人无直接的益处。如果您是学生，参加本实验能够帮您更直观地了解压力分布与肌电这种研究方法。

This research has potential contributions to science, especially cognitive neuroscience, but has no direct benefit to you personally. If you are a student, participating in this experiment can help you understand the research methods of pressure distribution and electromyography more intuitively.

9.报酬 Remuneration

50 CNY.

10.实验结果的反馈 Feedback of experimental results

如果对您对本研究感兴趣，可以留下联系方式给主试。本项研究结束时，我们会向您反馈本研究的主要结果。请注意，我们提供的结果是所有参与者的平均，我们不会向您提供您个人的研究数据。

If you are interested in this research, you can leave your contact information to the examiner. At the end of this research, we will give you feedback on the main results of this research. Please note that the results we provide are the average of all participants, and we will not provide you with your personal research data.

11. 您的权利 Your rights

- 您有权利获知本研究的相关信息及可能的风险和不适。
- 您自愿参加本实验；您可以随时中止参与实验并获得与参与时间相应的报酬。
- 您的数据会被以匿名方式处理，并不被用于本研究以外的其他目的。
- 您的个人隐私会受到保护。您的名字或者其他身份信息不会在任何发表物或教学材料中出现，除非得到您的书面授权。
- You have the right to be informed about the relevant information and possible risks and discomforts of this study.
- You voluntarily participate in this experiment; you can stop participating in the experiment at any time and get rewards corresponding to the time of participation.
- Your data will be processed anonymously and will not be used for purposes other than this research.
- Your personal privacy will be protected. Your name or other identifying information will not appear in any publications or teaching materials unless you have your written authorization.

12. 您的义务 Your obligations

- 科学是严肃的，请遵从任务指导语和主试的要求，认真完成实验任务。请关闭手机。
- 如果不明白实验任务的要求，请及时告诉主试。
- 如果您有不适于参加该实验的清况（如癫痫），请告知主试。
- 您在参加实验前不可使用药物或饮酒。
- 请不要触碰、移动或调节实验设备。
- 在任务进行过程中，请尽可能避免身体动作，以便我们能够获取可靠的研究数据。您的配合程度将决定实验的成败。
- Science is serious, please follow the task instructions and the requirements of the examiner, and complete the experimental tasks carefully. Please turn off your phone.
- If you do not understand the requirements of the experimental task, please tell the test supervisor in time.
- If you have any clear conditions that are not suitable for participating in the experiment (such as epilepsy), please inform the examiner.
- You must not use drugs or drink alcohol before participating in the experiment.
- Do not touch, move or adjust the laboratory equipment.
  During the task, please avoid body movements as much as possible so that we can obtain reliable research data. Your level of cooperation will determine the success or failure of the experiment.

13.联系方式

本知情同意书您可以保留一份副本。如果您有其他与本研究相关的问题，可以与 张玲燕 联系。

You may keep a copy of this informed consent. If you have any other questions related to this study, please contact Lingyan Zhang.

电话/Telephone：15869118003
Email: lingyanzh@outlook.com

14. 被试声明 Statement of subjects

我确认已经被告知本研究的目的、过程、可能的风险和不适，以及潜在的益处。我关心的所有问题都已得到满意的回答。我已经详细阅读了本《被试知情同意书》，明白自己的权利和义务。本人明白可以随时退出本研究而不会因此受到惩罚或遭受利益方面的损失。本人同意在浙江工业大学工业设计研究院参加本研究。

I confirm that I have been informed of the purpose, process, possible risks and discomfort, and potential benefits of this study. All my concerns have been satisfactorily answered. I have read the informed consent form of the subjects in detail and understand my rights and obligations. I understand that I can withdraw from this study at any time without any punishment or loss of interest. I agree to participate in this study in the Institute of industrial design, Zhejiang University of technology.

被试签名：王望诚 日期：2021.10.10

15. 主试声明 Statement of host

我已经向被试解释了研究的目的、研究的程序、潜在的危险和不适，以及她 / 他的权益。我已经尽最大可能回答了被试提出的与研究有关的问题。我声明将遵从肌电实验的标准作业程序。

I have explained to the subject the purpose of the study, the procedure of the study, the potential dangers and discomfort, and her / his rights and interests. I have tried my best to answer the questions related to the research. I declare that I will follow the standard operating procedures for the relevant experiments.

主试签名：[illegible] 日期：2021.10.10
